# Novel nesprin-1 mutations associated with dilated cardiomyopathy cause nuclear envelope disruption and defects in myogenesis

**DOI:** 10.1093/hmg/ddx116

**Published:** 2017-04-07

**Authors:** Can Zhou, Chen Li, Bin Zhou, Huaqin Sun, Victoria Koullourou, Ian Holt, Megan J. Puckelwartz, Derek T. Warren, Robert Hayward, Ziyuan Lin, Lin Zhang, Glenn E. Morris, Elizabeth M. McNally, Sue Shackleton, Li Rao, Catherine M. Shanahan, Qiuping Zhang

**Affiliations:** 1 King's College London British Heart Foundation Centre of Research Excellence, Cardiovascular Division, London SE5 9NU, UK; 2 Department of Cardiology, West China Hospital of Sichuan University, Chengdu 610041, China; 3 Laboratory of Molecular Translational Medicine; 4 Key Laboratory of Obstetric & Gynecologic and Pediatric Diseases and Birth Defects of Ministry of Education; 5 SCU-CUHK Joint Laboratory for Reproductive Medicine, West China Second University Hospital, Sichuan University, Chengdu, 610041, China; 6 Department of Molecular and Cell Biology, University of Leicester, Leicester LE1 9HN, UK; 7 Wolfson Centre for Inherited Neuromuscular Disease, RJAH Orthopaedic Hospital, Oswestry SY10 7AG, UK and Institute for Science and Technology in Medicine, Keele University, ST5 5BG, UK; 8 Center for Genetic Medicine, Northwestern University Feinberg School of Medicine, Chicago, IL 60611, USA

## Abstract

Nesprins-1 and -2 are highly expressed in skeletal and cardiac muscle and together with SUN (Sad1p/UNC84)-domain containing proteins and lamin A/C form the LInker of Nucleoskeleton-and-Cytoskeleton (LINC) bridging complex at the nuclear envelope (NE). Mutations in nesprin-1/2 have previously been found in patients with autosomal dominant Emery–Dreifuss muscular dystrophy (EDMD) as well as dilated cardiomyopathy (DCM). In this study, three novel rare variants (R8272Q, S8381C and N8406K) in the C-terminus of the *SYNE1* gene (nesprin-1) were identified in seven DCM patients by mutation screening. Expression of these mutants caused nuclear morphology defects and reduced lamin A/C and SUN2 staining at the NE. GST pull-down indicated that nesprin-1/lamin/SUN interactions were disrupted. Nesprin-1 mutations were also associated with augmented activation of the ERK pathway *in vitro* and in hearts *in vivo*. During C2C12 muscle cell differentiation, nesprin-1 levels are increased concomitantly with kinesin light chain (KLC-1/2) and immunoprecipitation and GST pull-down showed that these proteins interacted via a recently identified LEWD domain in the C-terminus of nesprin-1. Expression of nesprin-1 mutants in C2C12 cells caused defects in myoblast differentiation and fusion associated with dysregulation of myogenic transcription factors and disruption of the nesprin-1 and KLC-1/2 interaction at the outer nuclear membrane. Expression of nesprin-1α_2_ WT and mutants in zebrafish embryos caused heart developmental defects that varied in severity. These findings support a role for nesprin-1 in myogenesis and muscle disease, and uncover a novel mechanism whereby disruption of the LINC complex may contribute to the pathogenesis of DCM.

## Introduction

Dilated cardiomyopathy (DCM) is characterised by dilatation and impaired contraction of the left ventricle or both ventricles, and is an important cause of heart failure and sudden cardiac death, particularly in the young. The genetic causes of DCM are extremely complicated and over 50 genes have been implicated, many of them encoding components of the cytoskeleton and nuclear envelope (NE) (1,2).

Mutations in the *LMNA* gene, encoding the nuclear intermediate filament proteins lamin A/C, account for 6% of familial DCM patients in addition to causing a wide spectrum of diseases, named laminopathies. The laminopathies include Emery-Dreifuss muscular dystrophy (EDMD), which manifests with skeletal muscle wasting, heart conduction defects (CD) and DCM (3,4). Mutations in the *EMD* gene, encoding the inner nuclear membrane (INM) protein emerin, a binding partner of lamin A/C, also cause EDMD with CD (5). How mutations in both emerin and lamin A/C, which are ubiquitously expressed proteins, can lead to muscle-specific diseases has been a subject of debate for some time. However, evidence has shown that lamin A/C and emerin are both associated with the LInker of Nucleoskeleton and Cytoskeleton (LINC) bridge complex, which links the nucleus to the actin cytoskeleton. The major components of the LINC complex are: nesprins-1 and -2 (NE-spectrin repeat proteins) and SUN (Sad1p/UNC84)-domain containing proteins (SUN1/2) (6–8). Nesprin-1 and -2 can bind actin via a paired N-terminal Calponin Homology (CH) actin binding domains and the nuclear membrane via a C-terminal Klarsicht/ANC-1/Syne Homology (KASH) domain (9,10). The KASH domain also binds to SUN1/2 which span the INM and bind to lamin A/C directly via their N-terminal nucleoplasmic domain, thus providing a physical connection between the nucleus and the cytoskeleton (6,11,12). Mutations in nesprin-1 and -2 have been implicated in EDMD 4 (AD-EDMD 4, OMIM 612998) and 5 (AD-EDMD5, OMIM 612999), and SUN1 and SUN2 have also been implicated in EDMD (13–16). Thus, it has been suggested that disruption of the LINC complex by *LMNA* and *EMD* mutations, as well as *SYNE* (nesprin) and *SUN* mutations, may trigger effects on either chromatin structure causing deregulation of gene expression, or disruption of structural organisation of the cell, which is particularly important in muscle as it is subject to mechanical strain.

Another mechanism that may explain tissue specific disease is muscle-specific expression of LINC complex components. Alternative transcription and splicing of *SYNE1* and *SYNE2*, the genes encoding nesprin-1 and -2, generate multiple isoforms that vary greatly in size (17). The largest giant nesprin-1 and -2 isoforms localise at the outer nuclear membrane (ONM) and connect the nucleus to the actin cytoskeleton. However, smaller nesprin-1 and -2 isoforms, with a truncated N-terminus but sharing spectrin repeats (SRs) in common with the C-terminal regions of the giant proteins, also localise at the INM where they bind to emerin, lamin A/C and SUN1/2, forming a complex at the INM (18). These smaller isoforms, in particular, nesprin-1α_2_ and -2α_1_, are highly and specifically expressed in cardiac and skeletal muscle (7,19). Thus, mutations in *SYNE1/2* that disrupt these specific nesprin-1 and -2 isoforms, or mutations in other components of the LINC complex that disrupt their binding with nesprin-1 and -2, are likely to play a key role in muscle-specific laminopathies.

In support of this notion, mutations in the C-terminal regions of the nesprin-1 (*SYNE1*) and -2 (*SYNE2*) genes have been identified in patients with muscle specific disorders. This is in contrast to mutations located towards the N-terminus, which are associated with ataxia (13,14,20). For example, several missense mutations in the C-terminus were identified in several small family pedigrees, as well as in sporadic EDMD patients with DCM (13). Studies in fibroblasts from these patients showed abnormal localisation and binding of the LINC complex proteins lamin, emerin and SUN2, as well as nuclear morphology defects and loss of NE integrity. A missense mutation in the same C-terminal region of the *SYNE1* gene was identified in a DCM patient, which resulted in increased expression of nesprin-1 and lamin A/C, also indicating a perturbation of the LINC complex (14). Furthermore, nesprin-1 KASH domain knockout (KO) mice developed an EDMD-like phenotype and DCM (21), displaying muscle degeneration with elongated nuclei and reduced heterochromatin. Ablation of the C-terminal regions of both nesprin-1 and -2 in cardiomyocytes resulted in early onset cardiomyopathy (22). These mutant cardiomyocytes exhibited altered nuclear positioning, shape, and chromatin positioning, leading to impairment of gene expression in response to biomechanical stimuli due to loss of either nesprin -1 or -2 or both (22). Finally, in studies *in vitro*, overexpressing dominant-negative versions of nesprin KASH or SUN proteins in mouse C2C12 myoblasts perturbed the mechanical control of cell differentiation (23). In these studies, nuclear displacement and defects in nuclear rotation were also noted, implicating the LINC complex in myonuclear positioning.

Indeed, recent data have shown that the C-terminal region of nesprin mediates myonuclear positioning by attaching the microtubule (MT) network to the NE during embryonic muscle development and cell migration (24). These interactions occur via the MT motor proteins, dynein and kinesin, the latter being a heterotetramer of two kinesin heavy chain (KHC) subunits -Kif5A, Kif5B, or Kif5C- and two kinesin light chain (KLC)-1/2 subunits. Nesprin-2 was shown to interact with KLC-1/2 at the ONM via a newly identified four-residue tryptophan-acidic (W-acidic) ‘LEWD’ binding motif within an adaptive domain (AD) at its C-terminus, which is present in all muscle-specific isoforms (25–27). Disruption of the LINC complex with a dominant-negative nesprin-2 KASH impaired kinesin-1 association with the NE and induced nuclear aggregation in myotubes (24). As both nesprin-1 and -2 are highly conserved in this C-terminal region, it is likely that nesprin-1 may also mediate the nesprin/KLC interaction via the ‘LEWD’ binding motif but so far this has not been tested.

In this study, we screened the *SYNE1* and *SYNE2* genes in 218 DCM patients. We identified three novel nesprin-1 variants (R8272Q, S8381C, and N8406K) in seven patients and investigated their roles in NE organisation and myogenesis.

## Results

### Identification of three novel nesprin-1 mutants in DCM patients

Mutation screening of both the *SYNE1* and *SYNE2* genes was performed in 218 sporadic cases of DCM and 210 ethnically matched controls (Supplementary Material, Table S1). 23 exons for nesprin-1 corresponding to nesprin-1α_1_ and 1α_2_ and 16 exons for nesprin-2 corresponding to isoforms nesprin-2α, 2β and 2ɛ were screened. This screening strategy was based on high and specific expression of these isoforms in cardiac and skeletal muscle as well as mapping of the lamin A/C, emerin, SUN and KLC binding sites to domains within these isoforms (19,28–30).

**Figure 1:**
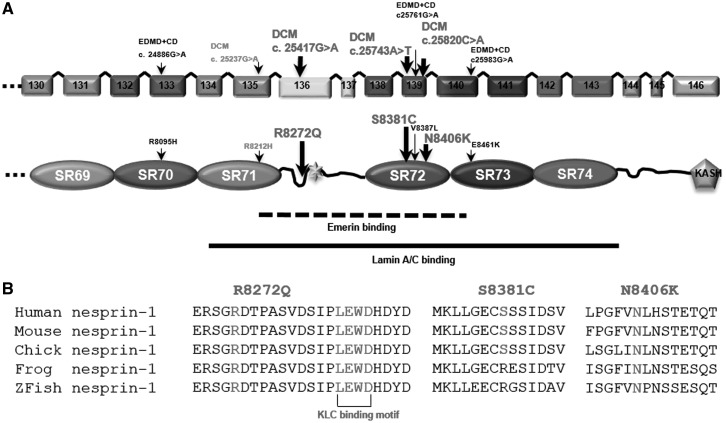
Identification of nesprin-1 variants in DCM patients. Mutation screening in *SYNE1* and *2* genes was performed in 218 DCM patients and 210 healthy controls, and identified seven patients harbouring three novel nesprin-1 mutations (R8272Q, S8381C, N8406K, shown in bold) in the C-terminus of nesprin-1 giant (**A**), equivalent to nesprin-1α, within an evolutionally conserved region containing the lamin and emerin binding domains (**A**, **B**). Previously identified nesprin-1 mutants in DCM (R8212H) and EDMD-CD patients (R8095H, V8387L and E8461K) were also shown (13,14). The KLC binding motif (LEWD) is shown in (B) (24,27).

Twelve single nucleotide polymorphisms (SNPs)/variants in *SYNE1* were identified, which included intronic sequence variations (*n* = 2), synonymous (*n* = 5) and non-synonymous, amino acid exchanges (*n* = 5). Eight of the variants were not present in 420 control alleles of an ethnically matched reference population (Supplementary Material, Table S2). No nesprin-2 SNPs/variants were identified in the region screened. Interestingly, three of the identified nesprin-1 mutations were unique DNA variants, which resulted in R8272Q, S8381C, N8406K amino acid exchanges in the C-terminus of the nesprin-1 giant as well as within the muscle specific isoform nesprin-1α_2,_ and identified in seven unrelated DCM patients (Fig. 1A, Supplementary Material). The amino acids changed by these missense mutations were in regions that were evolutionarily well conserved and within the mapped emerin and lamin A/C binding domains (25) and also the proposed KLC-1/2 binding domain in nesprin-1 (24,27) (Fig. 1B). The rarity of these variants and their conservation and positioning suggest they may be causative for DCM in the patients examined. ExAC database search revealed that population frequencies for the R8272Q allele were 0.0002145 (26/121204). Further *in silico* functional analyses showed that all three variants are predicted to cause significant functional impairment for nesprin-1 (Polyphen-2: possibly damaging, SIFT: damaging and Mutation Taster: disease causing). Therefore, all three rare variants were included in further cell biological investigations.

### Over-expression of nesprin-1 mutants disrupted nuclear morphology and reduced lamin A/C and SUN2 staining at the NE

**Figure 2:**
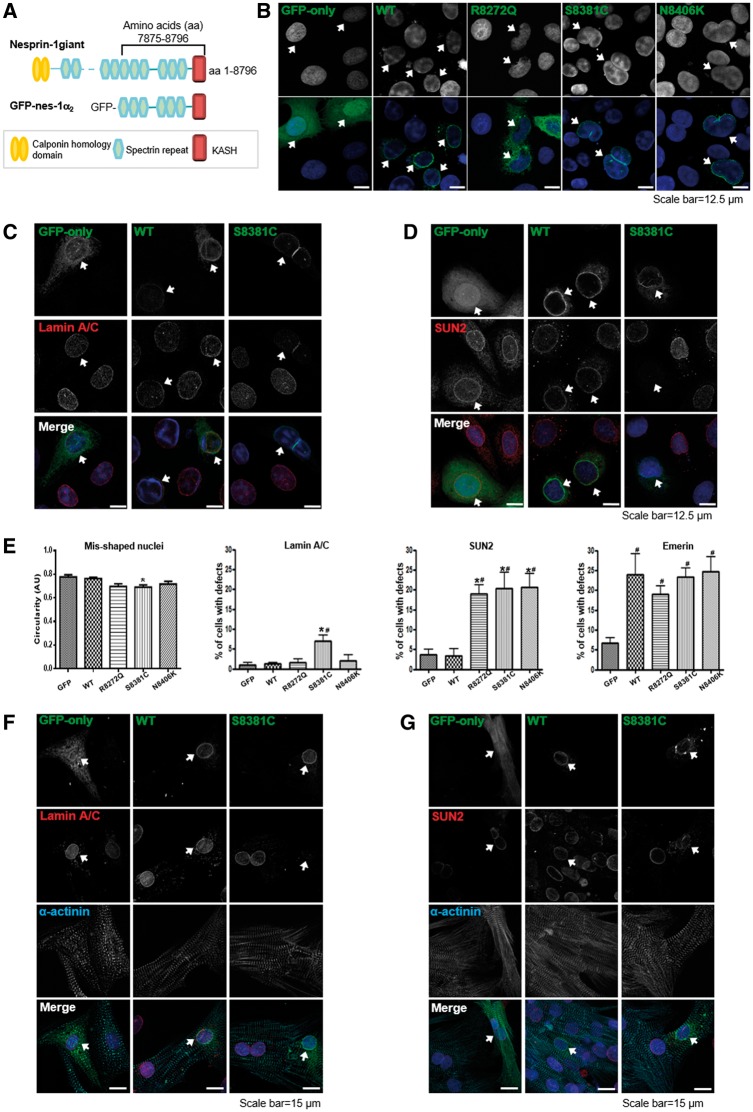
Overexpression of nesprin-1 mutants caused abnormal nuclear morphology and reduced NE staining of lamin A/C and SUN2. GFP-tagged nesprin-1α_2_ WT and mutants are shown schematically (**A**). The full length of this isoform is 977 amino acids, equivalent to amino acids 7875–8796 in nesprin-1 Giant. U2OS cells were transfected with either GFP-nesprin-1α_2_ WT or mutants. IF staining showed overexpression of mutants, especially S8381C, led to abnormalities in nuclear morphology (**B**), reduced lamin A/C and SUN2 staining at the NE (**C**, **D**, arrowed). The mis-shapen nuclei were measured by circularity, and mislocalisation of lamin A/C, SUN2 and emerin in the transfected cells was quantified by comparing the NE staining in non-transfected cells on the same slide. Graphical representation of the frequency of misshaped nuclei and defects in lamin A/C, SUN2, and emerin at the NE caused by all three mutants (**E**). At least 100 transfected nuclei were counted in more than three individual experiments and the results are presented as mean ± SEM and also analysed by Student’s *t*-tests and one-way ANOVA. (*P < 0.05 compared with WT; #: *P* < 0.05 compared with GFP only). In addition, IF showed both lamin A/C and SUN2 staining at the NE was reduced in the transfected NRCs using GFP-tagged nesprin1α_2_ mutant S8381C compared with GFP-tagged WT nesprin-1α_2_ (**F**, **G**, arrowed).

We generated GFP-tagged wild-type (WT) and mutant R8272Q, S8381C, N8406K constructs in the context of the muscle specific isoform nesprin-1α_2_ (Fig. 2A). When transfected into human osteosarcoma (U2OS) cells, abnormalities of nuclear morphology, measured by nuclei circularity, were induced by constructs harbouring the three novel nesprin-1 mutants when compared with WT (Fig. 2B and E). Immunofluorescence (IF) staining showed exogenous expression of the mutants, especially S8381C, caused weaker staining of lamin A/C at the NE (Fig. 2C and E and Supplementary Material, Fig. S1A), while all three mutants caused weaker staining of SUN2 at the NE, when compared with WT nesprin-1α_2_ (Fig. 2D and E and Supplementary Material, Fig. S1B). In addition, emerin was mis-localised by both the WT and mutants when compared with GFP alone (Fig. 2E). When the same constructs were transfected into neonatal rat cardiomyocytes (NRCs), IF staining showed that both lamin A/C and SUN2 staining was weaker at the NE with all three mutants compared with WT nesprin-1α_2_ (Fig. 2F and G and Supplementary Material, Fig S1C and D), indicating nesprin-1 mutants differentially affect the localisation of NE binding partners.

### Nesprin-1 mutants disrupt the interactions between nesprin-1, lamin A/C and SUN2 within the NE complex

**Figure 3:**
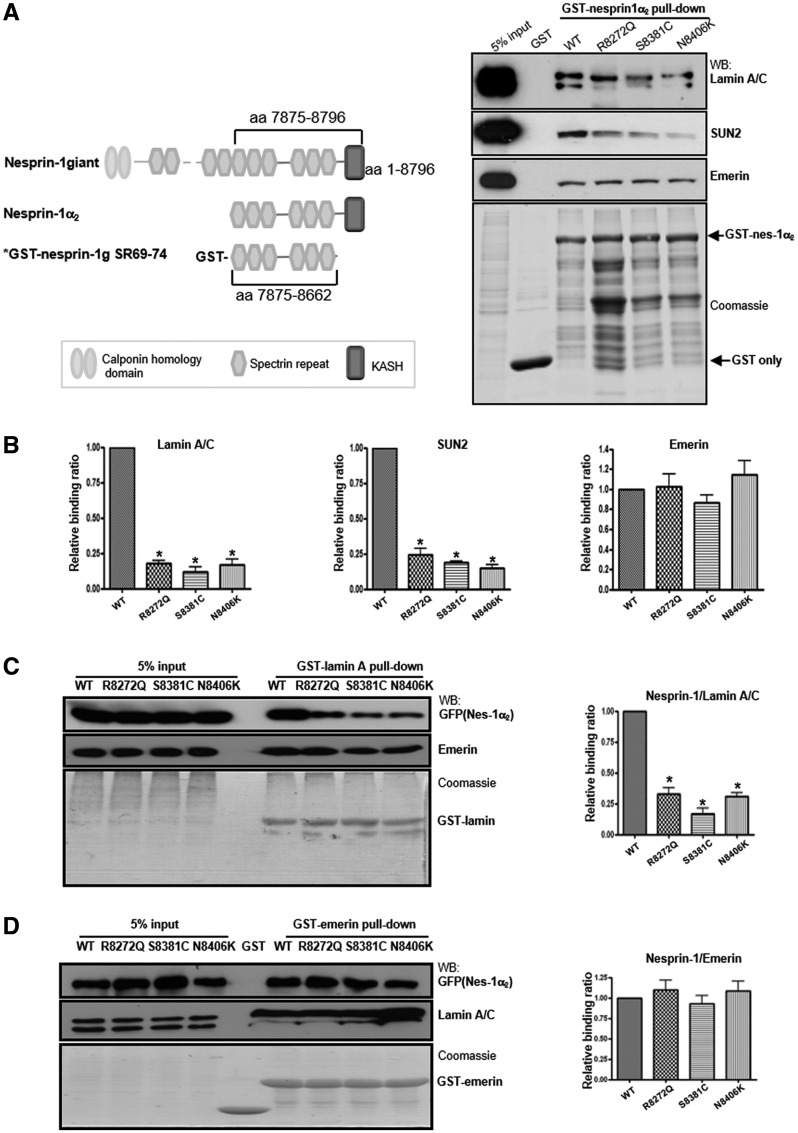
Nesprin-1 mutants affect the interaction between nesprin-1α_2_ and lamin A/C or SUN2. GST-tagged WT nesprin-1α_2_ SR1-6 (equivalent to nesprin-1 giant SR 69–74) and mutants are generated and shown schematically (3A left panel, labelled with *), which constructs consisted of 837 amino acids, lacking KASH domain, equivalent to amino acids 7875–8662 of nespin-1 giant. GST pull-down using either GST-WT or mutant nesprin-1 beads showed all three mutants affected binding between nesprin-1 and lamin A/C or SUN2, but not emerin (**A**, **B**), which was confirmed by reverse GST pull down by transfecting either GFP-nesprin-1α_2_WT or each mutant and using either GST-lamin A (amino acids 356–665) (**C**) or GST-emerin (amino acids 1–176) beads (**D**). The binding for each mutant was quantified by densitometry with respect to the input material and expressed as a ratio of the value obtained for WT protein. Three independent experiments were performed shown as mean ± SEM, *P < 0.05 using one-way ANOVA analysis. Coomassie blue staining gel also showed equal amount of GST-nesprin 1α_2_, lamin or emerin beads used.

To investigate whether the mutants affected the binding between nesprin-1 and its NE binding partners, GST-tagged WT and mutant nesprin-1α_2_ constructs lacking the KASH domain (equivalent to nesprin-1 giant amino acids 7875–8662) were generated (Fig. 3A left panel). Protein lysates from un-transfected U2OS cells and transfected cells with Myc-SUN2 (full length) were subjected to GST pull-down using either WT or mutant GST-nesprin-1α_2_. All three mutants had significantly reduced binding to lamin A/C and SUN2, but not emerin (Fig. 3A right panel, and B) when compared with WT nesprin-1α_2_. A reverse GST pull-down was also performed. Protein lysates from U2OS cells transfected with either GFP-nesprin-1α_2_ WT or each of three mutants were subjected to pull-down using either GST-lamin A (amino acids 356–665, containing nesprin-1/2 binding domain) or GST-emerin (amino acids 1–176, lacking the TM domain). The results confirmed all three mutants had significantly reduced binding to lamin A (Fig. 3C), but not emerin (Fig. 3D). Taken together, these data indicated that these nesprin-1 mutations in the 1α_2_ region cause disruption of the lamin A/C, SUN2 and nesprin-1 complex at the INM.

### Nesprin-1 mutants augment activation of extracellular signal-regulated kinase (ERK) pathway

**Figure 4:**
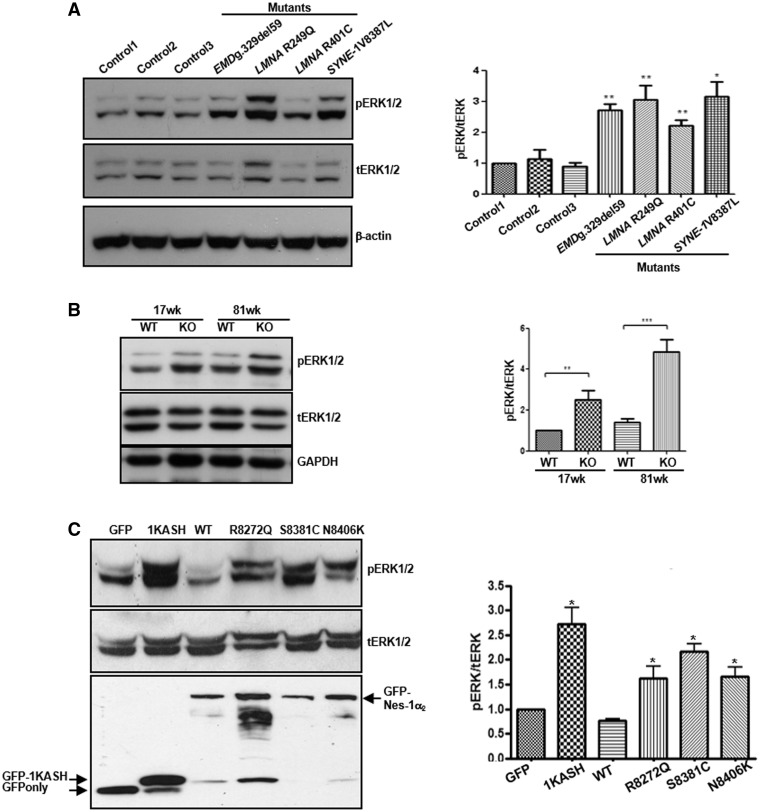
Nesprin-1 mutants cause aberrant activation of MAPKs. WB showed that aberrant activation of pERK was observed in human dermal fibroblasts from EDMD-DCM patients carrying nesprin-1 mutation (V8387L), lamin A/C (R249Q and R401C) and emerin (g.329del59) mutations (**A**), as well as nesprin-1 KASH KO mice heart collected at 17 and 81 weeks, respectively (**B**). WB also showed overexpression of all three nesprin-1 mutants and dominant negative-1KASH led to augmented pERK activity compared with WT nesprin-1 and GFP alone (**C**). GFP empty vector was used for negative control and a dominant negative-1KASH construct as a positive control. Three independent experiments were performed shown as mean ± SEM, *P < 0.05 using one-way ANOVA analysis.

Previous studies have shown that cells harbouring lamin and emerin mutations have altered activation of ERK1/2 (31,32). To investigate whether nesprin-1 defects lead to aberrant ERK1/2 activation, protein lysates from human dermal fibroblasts (HDFs) derived from EDMD and DCM patients carrying either nesprin-1, lamin A/C or emerin mutations (13), and heart tissue from WT and nesprin-1 KASH KO mice with EDMD-like phenotype and DCM (21) were examined. Western blot (WB) showed significant up-regulation of ERK1/2 activity in patient fibroblasts (Fig. 4A) and nesprin-1 KASH KO mouse hearts (Fig. 4B). To determine whether the novel nesprin-1 mutants could also induce activation of ERK1/2 signalling, GFP-nesprin-1α_2_ WT and mutants, as well as a dominant-negative nesprin-1 KASH (1KASH), which has previously been shown to displace endogenous nesprin-1 and cause NE defects (6,33), were transiently transfected into C2C12 and H9C2 myoblasts. Immunoblotting with antibodies against phosphorylated ERK1/2 (pERK) and total ERK1/2 (tERK) as well as phosphorylated ELK1 (pELK1), a downstream target of ERK1/2, demonstrated that overexpression of the GFP tagged-nesprin-1 mutants and 1KASH increased the amount of pERK1/2 and pELK1 compared with GFP alone or WT nesprin-1α_2,_ in both C2C12 cells (Fig. 4C) and H9C2 cells (Supplementary Material, Fig. S2).

### Nesprin-1 mutants lead to dysregulation of myogenic transcription factors

**Figure 5:**
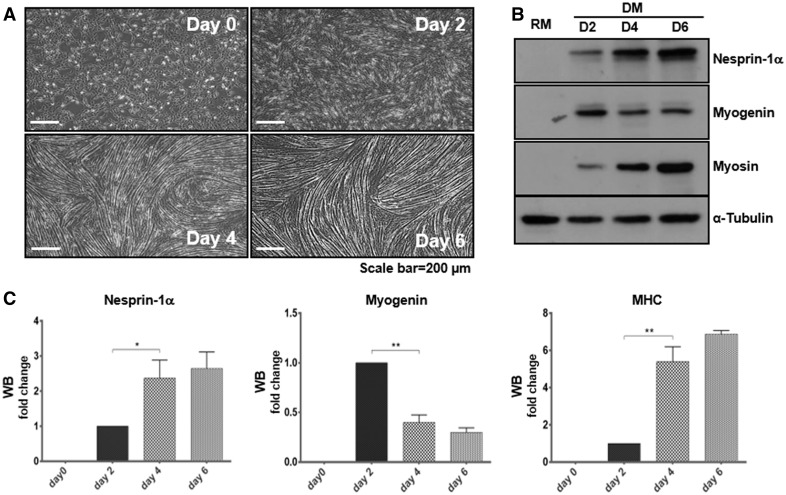
Expression level of nesprin-1α increase during C2C12 myoblast differentiation. C2C12 myoblasts in regular medium (RM) were stimulated with a low serum differentiation medium (DM), leading to myotube formation during the process (**A**). WB showed endogenous expression levels of nesprin-1, myogenin and myosin/MHC increased during myotube formation (**B**, **C**). Nesprin-1α protein levels (detected by MANNES1E) were highest at day 6, whereas myogenin was highest at day 2, and MHC was detected from day 2 and increased until day 6.

To further investigate if these three novel mutants cause muscle cell dysfunction, we next used the C2C12 mouse myoblast differentiation model. Previous studies showed that mutations in *LMNA* and overexpression of 1KASH or SUN proteins in C2C12 cells disrupt myoblast differentiation (23,34,35). Myogenesis is driven by myogenic transcription factors (MTFs) such as MyoD and myogenin (36,37). To determine the expression levels of endogenous nesprin-1α, MyoD and myogenin as well as the skeletal muscle specific protein myosin heavy chain (MHC) during the differentiation process, protein lysates were collected at days 0, 2, 4 and 6 following induction (serum withdrawal). WB and/or qPCR showed that endogenous expression levels of nesprin-1α (including both 1α_1_ and 1α_2_), MyoD, myogenin and MHC were increased along with myotube formation (Fig. 5A–C, Supplementary Material, Fig. S3). Nesprin-1α levels peaked at day 6, whereas MyoD and myogenin levels peaked at day 2. MHC was detected from day 2 and increased until day 6. Therefore, day 2 for MyoD and myogenin, and day 6 for MHC were chosen as the optimised investigation time points for early and late differentiation in further experiments.

**Figure 6:**
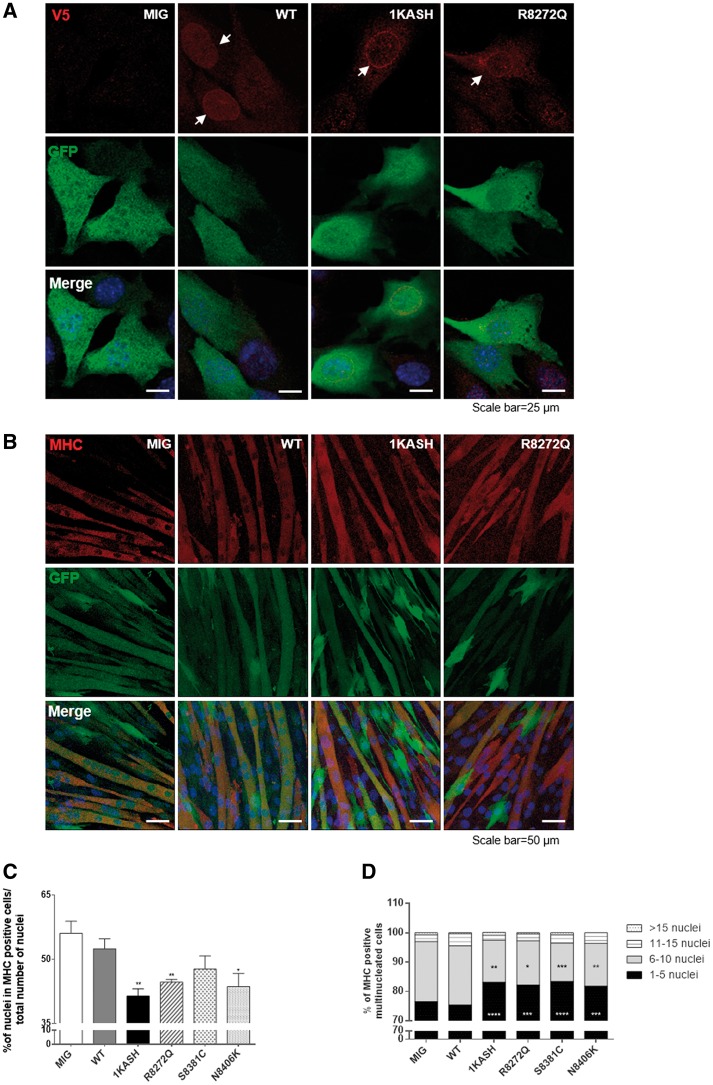

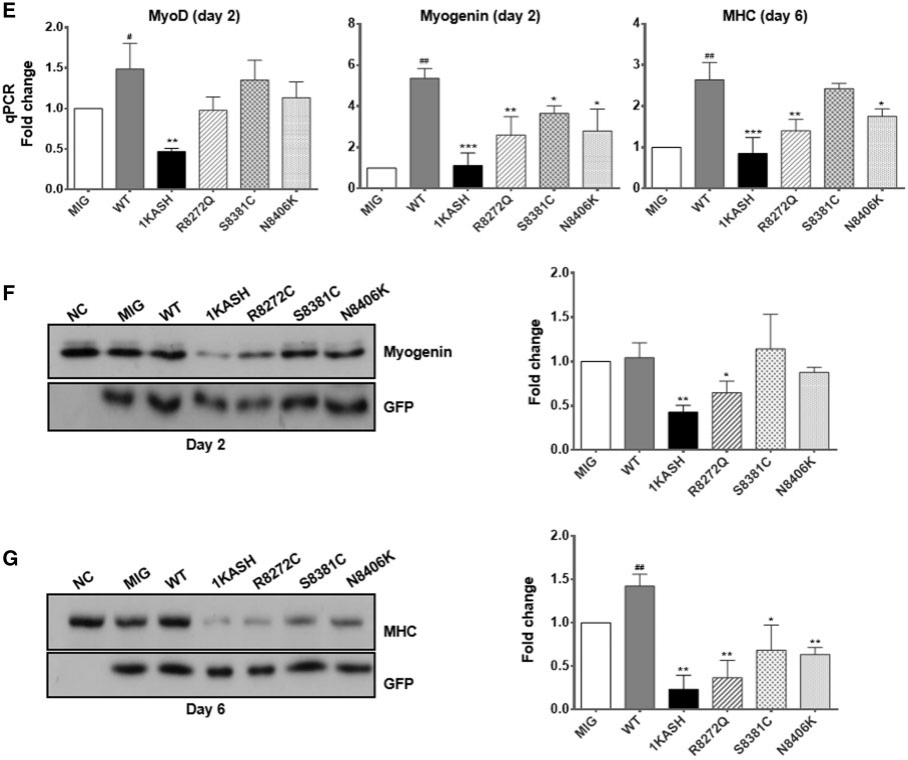
Nesprin-1 mutants cause defects in myoblast differentiation. IF showed that exogenously expressed V5-tagged WT-1α_2_ or mutants and dominant genative-1KASH were localized at the NE. GFP was expressed as a reporter for retroviral infection (**A**). Upon differentiation, fewer multinucleated myotubes were observed in cells transduced with mutant R8272Q and dominant negative-1KASH compared with C2C12 cells transduced with eGFP alone (MIG only) or nesprin-1α_2_ WT (**B**). The fusion index was reduced in cells transduced with the mutant R8272Q and dominant negative-1KASH compared with 1α_2_ WT (**C**), more than 600 nuclei for each clone were counted by microscopy (63× objective) at day 6, three independent experiments were performed for each clone. Further analysis of the MHC positive multinucleated populations revealed that myotubes expressing nesprin-1 mutants contained fewer nuclei compared to the controls (**D**). qPCR and WB showed that nesprin-1 mutant R8272Q and dominant negative-1KASH caused significant reduction of myogenin (**E**, **F**) and MHC (E, **G**) levels at DM day 2 and day 6 respectively, qPCR also showed that dominant negative-1KASH caused significant reduction of MyoD (E) levels at DM day 2. All were normalized to GFP, three independent experiments were performed shown as mean ± SEM, *P < 0.05 using Student’s *t*-tests or two-way ANOVA analysis.

To investigate whether the novel nesprin-1 mutants could disrupt muscle cell differentiation, we used retroviral transduction to generate C2C12 cells stably expressing V5-tagged nesprin-1α_2_ WT and mutant constructs as well as dominant negative-1KASH, which were cloned into a MIG (IRES-GFP) retroviral vector. GFP protein was expressed independently as a reporter for retroviral infection. Fluorescence-activated cell sorting (FACS) was performed to purify the infected GFP positive populations and these were used in subsequent muscle cell differentiation experiments (Supplementary Material, Fig. S4A, B). IF staining showed that exogenously expressed V5-tagged WT nesprin-1α_2_, mutants and 1KASH were observed at the NE in myoblasts (Fig. 6A, Supplementary Material, Fig. S5A). Efficient differentiation of C2C12 cells transduced with eGFP alone (MIG only) or nesprin-1α_2_ WT was observed, with many multinucleated myotubes formed within 5–6 days. However, fewer myotubes were formed in the cells transduced with either nesprin-1α mutants or 1KASH (Fig. 6B, Supplementary Material, Fig. S5B). Quantification of the fusion index i.e. the percentage of nuclei incorporated into MHC positive multinucleated cells vs. the total number of nuclei, showed a significant reduction in cells transduced with the mutants, in particular, R8272Q and N8406K as well as 1KASH when compared to WT (Fig. 6C). Further analysis of the MHC positive populations revealed that myotubes expressing nesprin-1 mutants contained fewer nuclei compared to the controls (Fig. 6D), indicating a defect in myoblast fusion. Both qPCR and WB showed that the levels of myogenin and MHC were dramatically reduced in cells transduced with the mutants, particularly R8272Q and 1KASH when compared with nesprin-1α_2_ WT at day 2 and 6 respectively (Fig. 6E–G). qPCR also demonstrated that MyoD was significantly reduced in the cells transduced with 1KASH at Day 2 (Fig. 6E).

### Nesprin-1 mutants cause disruption of nesprin-1 and KLC interaction

**Figure 7:**
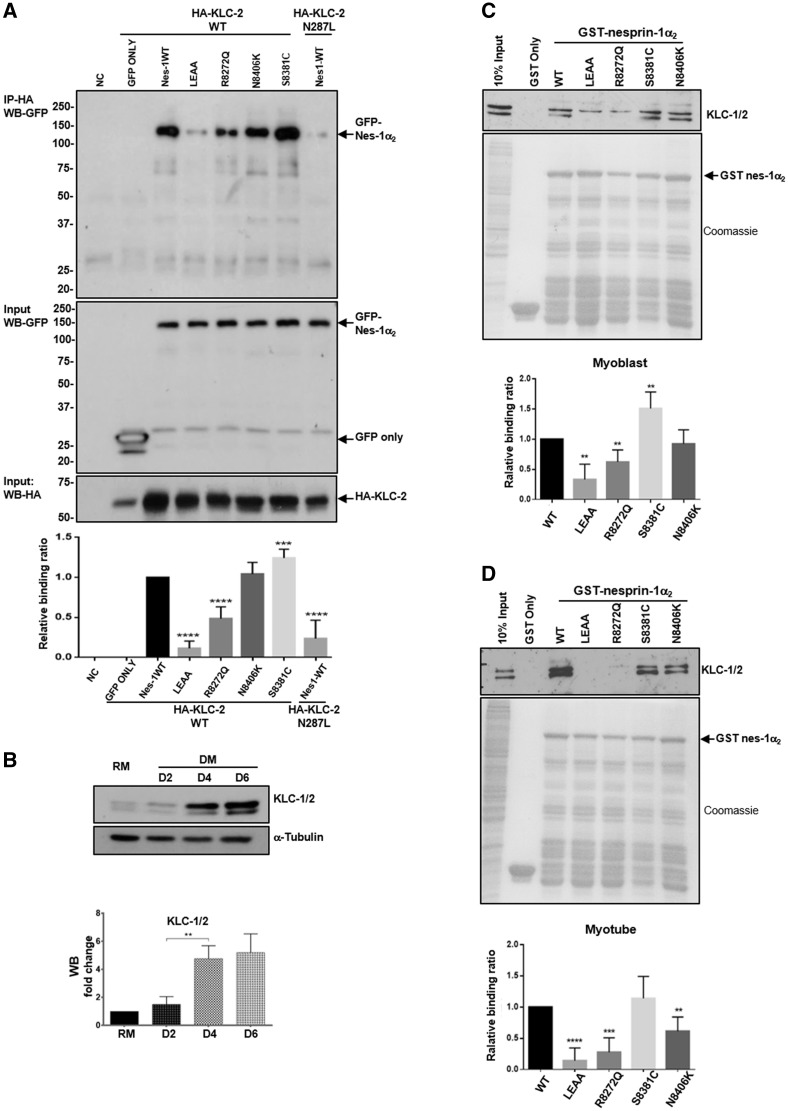
Defects in nesprin-1 and KLC-1/2 interaction. Overexpression and IP showed the binding between nesprin-1WT/mutants and KLC WT/mutants in U2OS cells, HA-KLC2-N287L was co-expressed with GFP-nesprin-1α_2_ WT as a positive control, GFP empty vector with HA-KLC-2 WT as a negative control (**A**). WB showed endogenous expression levels of KLC-1/2 increased during myotube formation (**B**). GST pull-down showed the binding between nesprin-1 and KLC-1/2 using either GST-WT or mutant nesprin-1 beads in myoblasts (C) and myotubes (**D**), respectively. The binding for each mutant was quantified by densitometry and expressed as a ratio of the value obtained for WT protein. Three independent experiments were performed shown as mean ± SEM, *P < 0.05 using Student’s *t*-tests.

Recent data showed that nesprin-2 interacts with KLC-1/2 via a conserved ‘LEWD’ motif at the C-terminus of nesprin-2 (27). This motif is also present in nesprin-1 (Fig. 1B) (25–27), suggesting nesprin-1 may also bind to KLC-1/2 and be involved in connecting the nucleus to the microtubule network and therefore play a role in myonuclear positioning. By treatment of C2C12 cells expressing the plasmid containing V5-tagged nesprin-1α_2_ WT with digitonin or NP-40, we showed that nesprin-1α_2_ was also localised at the ONM in addition to its previously identified INM localisation where it co-localises and binds to lamin A/C (Supplementary Material, Fig. S6). Therefore, we set out to investigate whether nesprin-1 could also bind to KLC-1/2. GFP-1α_2_ WT or nesprin-1α_2_-LEAA (mutated WD/AA within the LEWD motif) were co-expressed with Flag-tagged KLC-2 WT (a dominant light chain isoform in muscle) or the KLC-2 N287L mutant, previously shown to disrupt nesprin-2/KLC binding (27) in U2OS cells. Immunoprecipitation (IP) and WB showed that nesprin-1α_2_ could efficiently bind to KLC-2, and this binding was disrupted by either mutation (LEAA) in the conserved nesprin-1 LEWD motif or the KLC-2 N287L mutant (27) (Fig. 7A). We then tested the nesprin-1 mutants using this system and found that the R8272Q mutant had significantly reduced binding to KLC-2 compared with nesprin-1α_2_ WT, while the S8381C mutant appeared to enhance KLC-2 binding (Fig. 7A).

**Figure 8:**
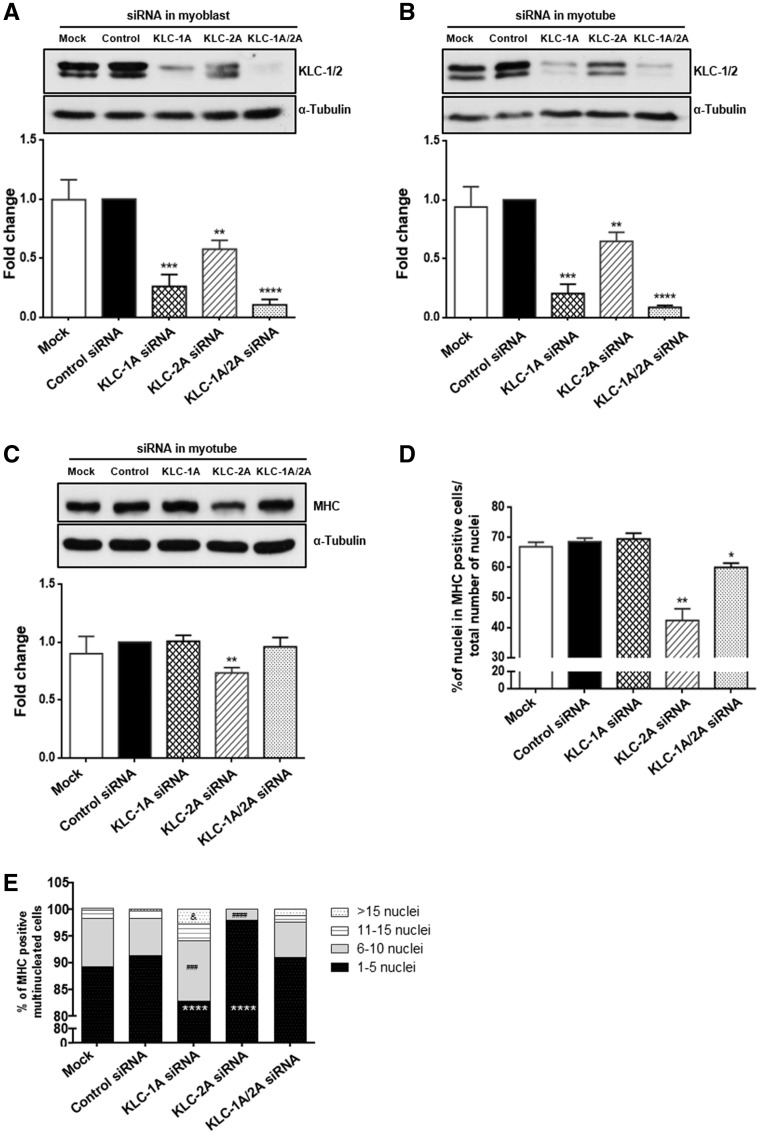
siRNA knockdown of KLC-1/2 cause defects in myoblast fusion and differentiation. WB showed the expression levels of both KLC-1 and -2 were reduced in myoblasts (**A**) and myotubes (**B**) upon KLC-1/2 depletion. The expression level of MHC (**C**) and the fusion index (**D**) were significantly reduced in myotubes especially upon KLC-2 depletion (using KLC-2A oligo), more than 800 nuclei for each clone were counted by microscopy (63× objective) at day 6, three independent experiments were performed for each clone. Further analysis of MHC positive multinucleated cells revealed that KLC-2 depletion resulted in fewer nuclei per myotube. In contrast, KLC-1 depletion (using KLC-1A oligo) led to significantly more clustered nuclei per myotube when compared with controls (**E**). Means and SEM were obtained from three independent experiments for each clone. *P < 0.05 using Student’s *t*-tests or two-way ANOVA analysis.

Next we tested whether these mutants affect nesprin-1/KLC-1/2 binding during muscle cell differentiation. Firstly, WB showed that, similar to nesprin-1α, the endogenous expression level of KLC-1/2 increased during myoblast differentiation (Figs. 5B and7B). Using GST-nesprin-1α_2_ WT (lacking KASH domain), nesprin-1α_2_-LEAA and the three mutants, pull-down further confirmed that nesprin-1α_2_ efficiently binds to KLC-1/2, and this binding was disrupted by the mutated LEWD motif-LEAA. In addition, the R8272Q mutant had significantly reduced binding to KLC-1/2 compared with WT in C2C12 cells. This reduced interaction was especially evident in myotubes where expression of these two proteins is highest (Fig. 7C and D). Finally, we tested whether depletion of KLC-1/2 using multiple siRNA oligos would affect fusion of myoblasts and differentiation in a similar manner to that observed in the R8272Q mutant. WB showed the expression levels of both KLC-1 and -2 were reduced in both myoblasts and myotubes after KLC-1/2 depletion (Fig. 8A and B, Supplementary Material, Fig. S7C and D), and IF also showed a reduced KLC-1/2 staining at the NE, however, nesprin-1 remained at the NE (Supplementary Material, Fig. S7A). Moreover, MHC levels and the fusion index were significantly reduced in myotubes upon KLC-2 knockdown (Fig. 8C and D, Supplementary Material, Fig. S7E and F). Further analysis of MHC positive multinucleated cells (myotubes) revealed that KLC-2 depletion resulted in fewer nuclei per myotube (Fig. 8E, Supplementary Material, Fig. S7G), indicating a defect in myoblast fusion. In contrast, KLC-1 depletion led to significantly more clustered nuclei per myotube when compared with controls (Fig. 8E, Supplementary Material, Fig. S7B), indicating a defect in nuclear positioning in addition to myoblast fusion.

### Human nesprin-1α_2_ WT causes heart developmental and conduction defects in zebrafish embryos while mutants induce a less severe heart phenotype

**Figure 9:**
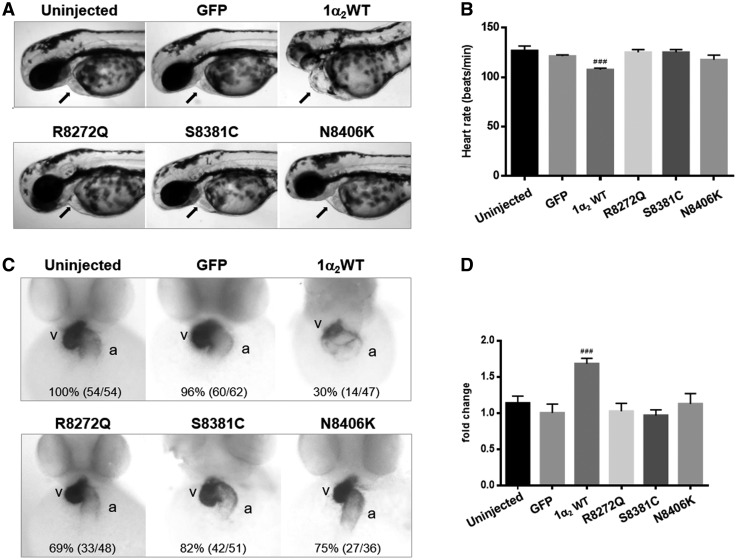
Human nesprin-1α_2_ WT induces heart development defects in zebrafish. Lateral views of zebrafish live embryos at 48 hpf. The pericardium (**A**, arrowed) and heart rate are shown for each corresponding mRNA injected (**B**), zebrafish embryos with nesprin1α_2_ WT mRNA showed slow heart rate and dilated atrial chambers. Whole-mount *in situ* hybridization (WISH) monitoring expression of the *myl7* gene at 48 hpf (**C**), the numbers (left in brackets) indicate the percentage of embryos displaying the phenotype represented in the picture shown, the numbers (right in brackets) is the total numbers counted of observed embryos. The relative atrium area of embryos for the corresponding mRNA injection was measured and calculated by the area of *myl7* expression using ImageJ (**D**), which was normalised to the atrium area of the embryos injected with GFP mRNA. Embryos are in ventral views with the anterior at the top. About 4–7 embryos for each injection were measured. Means and SEM were obtained from three independent experiments for each treatment. *P* < 0.001 using Student’s *t*-tests. a: atrium, v: ventricle.

To investigate whether the nesprin mutants affect cardiac structure or function *in vivo*, we generated a zebrafish model by expressing nesprin-1α_2_ WT and mutants (R8272Q, S8381C and N8406K) in zebrafish embryos via injection of the corresponding human *SYNE1α_2_* (nesprin-1α_2_) mRNAs at the one-cell stage. At 48 h post-fertilization (hpf), zebrafish embryos expressing human nesprin-1α_2_ WT showed heart defects including dilated atrial chambers with reduced heart rate (Fig. 9A and B, Supplementary Material, video). There was also evidence of abnormal anterior-posterior axis development and curved tails (Supplementary Material, video). Furthermore, whole-mount *in situ* hybridization (WISH) demonstrated that the expression of myosin light chain polypeptide 7 (*myl7*), the ortholog of the human regulatory myosin light chain (RLC) gene (38,39), was reduced in 30% of the embryos expressing human nesprin-1α_2_ WT. The expression pattern of this gene also highlighted the dilated atrial chambers and heart developmental defects such as abnormal looping (Fig. 9C and D;Supplementary Material, Fig. S8A and B). In contrast, expression of the human nesprin-1α_2_ mutants induced some heart developmental defects, such as abnormal heart looping and malpositioning of heart chambers (Supplementary Material, Fig. S8A and B), but without heart enlargement or heart rate defects. There was also evidence of abnormal anterior-posterior axis development in all the embryos, when compared with uninjected and GFP expressed embryos (Supplementary Material, Fig. S8C and D). Taken together these zebrafish larvae data suggests that overexpression of human nesprin-1α_2_ WT leads to cardiac developmental defects while nesprin-1α_2_ mutants induced a less severe heart phenotype.

## Discussion

Mutations in lamin A/C and emerin, which form a complex at the INM, can cause EDMD with conduction system defects, as well as DCM. Similarly, missense mutations in the C-terminal region of the nesprin-1 (*SYNE1*) and -2 (*SYNE2*) genes and *SUN1/2* genes were identified in EDMD-DCM patients, leading to disrupted nesprin/lamin/emerin/SUN1/2 interactions and nuclear morphology defects (40,41). In the current study, three novel missense mutations (R8272Q, S8381C and N8406K) were identified in the same C-terminal region of nesprin-1 in DCM patients, which affected nuclear morphology and impaired protein–protein interaction with lamin A/C and SUN2. The mutants also augmented ERK activation *in vitro*. Importantly, these mutations, especially mutant R8272Q, caused defects in myoblast differentiation associated with dysregulation of MTFs and disruption of the nesprin-1/KLC-1/2 interaction at the ONM. These findings provide new information on how nesprin-1 performs multi-functional roles in muscle cell development and disease via its connections at both INM and ONM, and also how these mutations and disruption of the LINC complex contribute to muscle dysfunction, and potentially the pathogenesis of DCM.

### Nesprin-1 mutations interfere with NE organisation

Mutations in the *LMNA* gene, encoding A-type lamins, cause a number of different tissue specific laminopathies, including DCM (42). The molecular pathogenesis of these diseases is unknown but in some cases may be related to disruption of the LINC complex. Mice lacking A-type lamins display phenotypes that are reminiscent of human muscular dystrophies and cardiomyopathies (43,44). Embryonic fibroblasts derived from *LMNA* KO mice are characterised with grossly misshapen cell nuclei and a structurally weakened NE, and mislocalization of nesprins leading to reduced mechanical stiffness (45). Mutations in both nesprins and SUN1/2 have also been implicated in EDMD-DCM, whereby mutants cause abnormal localisation of the LINC complex proteins including lamin, emerin and SUN, and disrupted nesprin/lamin/emerin/SUN interactions, leading to defects in nuclear morphology and nuclear-cytoskeletal coupling (13–16). Furthermore, the disruption of endogenous LINC complexes by recombinant dominant negative-KASH constructs of nesprins-1, -2 and -3 also causes a significant loss of mechanical stiffness (6,33,46). Interestingly, the three novel missense mutations (R8272Q, S8381C and N8406K) identified in DCM patients in this study reside within the unique AD region at the C-terminus of the nesprin-1 and -2 proteins (25,26). This region is evolutionally highly conserved and overlaps with the lamin and emerin binding regions. It has relatively little secondary structure and is likely to be flexible (26), and potentially able to adapt its conformation to stabilise the associations between nesprin SRs, lamin A/C and emerin at the INM (for nesprin-1α_2_) or SUN proteins at the ONM (for nesprin-1α_2_ and -1 giant isoforms). The three nesprin-1 mutations (R8272Q, S8381C and N8406K) identified in this study cause changes in the charge or hydrophilic/hydrophobic properties of conserved amino acids and likely affect the structure/flexibility of the AD region of nesprin-1, altering it interactions with lamin A/C and SUN2. These binding changes were most likely due to disruption of the functions of all the endogenous nesprin-1 isoforms containing the KASH domain in the transfected cells (19). Interestingly, the R8272Q mutant was able to bind lamin A at comparable levels to the WT nesprin-1, in contrast to weak binding to lamin C. Our current understanding of the interactions between nesprins and lamin A and lamin C remains incomplete, so this apparent binding difference requires further investigation. We did not observe a defect in binding between nesprin-1 mutants and emerin, which is consistent with the notion that mutations in emerin are primarily associated with EDMD rather than DCM (28,47). The data above support possible involvement of LINC complex disruption in the pathogenesis of DCM. Future studies on structure/flexibility of the SRs that are predominantly present in the muscle-specific nesprin isoforms will be required to confirm this hypothesis.

Several studies on *LMNA* KO and H222P knock-in mice have indicated activation of MAPKs in the development of DCM. Inhibitors of MAPKs could partially rescue the DCM phenotype, implicating ERK in the pathogenesis of lamin A/C cardiomyopathy (48,49). Our data showed that enhanced ERK activity is also observed in heart tissue from the nesprin-1 KASH KO mice, fibroblasts derived from EDMD-DCM patients and nesprin-1 mutant transfected cells, suggesting that nesprin and lamin A/C function through a similar pathway and trigger up-regulation of ERK. Future work will examine how NE proteins regulate ERK activity potentially via influencing interactions between A-type lamins, NE proteins and components of MAPK cascades, as well as nuclear translocation of activated MAPKs (50,51).

### Nesprin-1 mutations and dysregulation of MTFs in myoblast differentiation

Nesprin-1α_2_ is highly expressed in both skeletal and cardiac muscles as previously shown (7,52,53). Although there were no obvious muscle phenotypes recorded, it is plausible that in the patients presenting with the *SYNE1* mutations the skeletal muscle dysfunction was too subtle or underestimated at clinical examination. Alternatively, in addition to these *SYNE1* mutations, another cardiac disease gene is mutated thus enhancing the phenotype. Therefore, due to restricted accessibility to patient samples, we focused on investigating if these three novel mutants cause muscle dysfunctions by using the C2C12 mouse myoblast differentiation model.

Myogenesis involves a series of sequential steps. Myoblasts originating from the mesoderm are converted to skeletal muscle lineage myoblasts after MyoD expression, enter the cell cycle and proliferate, then withdraw from the cell cycle and initiate differentiation with expression of MTFs such as myogenin (54). Next cell fusion occurs to form multinucleated myotubes and expression of the muscle specific protein MHC. Increasing evidence indicates that multiple cell signalling pathways play critical roles in myoblast fusion, including those involved in cytoskeleton organization, cell adhesion and migration (55) as well as extracellular signalling molecules and components of extracellular matrix (54,56). Recent literature shows that LINC complex components, including nesprins and SUN1/2, mechanically couple the nucleus to the extracellular matrix and play an important role in differentiating muscle. In addition, these proteins have been implicated in regulating chromatin structure and gene expression. For example, C2C12 cells expressing a *LMNA* R453W mutant have reduced capacity to differentiate, yet retain an unaltered morphology (57), suggesting that subtle abnormal changes in lamina structure or composition may impair the anchoring of chromatin to the NE, affecting gene regulation and perturbing myogenesis.

Using retroviral transduction in mouse C2C12 myoblasts in the current study, our data showed myoblasts expressing mutant nesprin-1α_2_ R8272Q or dominant negative-1KASH had a significantly lower capacity to differentiate and form multinucleated cells than those expressing 1α_2_ WT, although exogenously expressed 1α_2_ WT and mutants were observed to be properly localised at the NE. Importantly, the inhibition of multinucleation by dominant negative-1KASH and 1α_2_ mutants, especially R8272Q, is potentially linked to the inhibition of myogenin and MHC expression in the transduced C2C12 cells. Our data also showed that nesprin-1α_2_ was expressed in the initial stage crucial for converting C2C12 myoblasts into myotubes and promoted myoblast differentiation during the process of myotube formation, whereas the mutants reduced or abolished the effects, suggesting that expression of nesprin-1α_2_ mutants does not alter the proliferation of myoblasts, but rather impairs their capacity to express muscle-specific genes (myogenin and MHC). This results in an inability to fuse, especially observed in the R8272Q mutant, which also explains why nesprin-1 mutants affect the expression levels of myogenin more severely than MyoD, because MyoD is already expressed prior to initiation of differentiation at a stage when the expression level of nesprin-1 is still low. Therefore, we propose the following mechanisms whereby nesprin mutants may affect muscle differentiation: regulation of myogenin and expression of MHC are influenced by the LINC complex; nesprin mutants may fail to build a functional scaffold and/or to maintain chromatin compartmentalisation with lamin A/C, leading to an alteration in the amount of heterochromatin formed and/or its localisation. Potentially this causes defects in initiation of the terminal differentiation process resulting in decreased or delayed expression of myogenin and MHC, thus causing a delayed differentiation. This effect may be similar to that observed in C2C12 cells expressing an *LMNA* R453W mutant (57) that showed a reduced capacity to differentiate. However, further experimentation on how the nesprin-1 mutants affect regulation of MTFs is required to elucidate the precise function of nesprin in muscle cell differentiation.

### Nesprin-1 mutations disrupt nesprin-1/KLC interaction and myoblast fusion

Proper nuclear positioning and movement is critical in muscle cell differentiation and development. In the nucleus, changes in gene activity that occur during terminal cell differentiation are correlated with gene migration and relocation (58,59). Nuclear movement and positioning are driven by cytoskeletal networks of MTs, actin and/or intermediate filaments and involve a connection between the cytoskeleton and the NE, mediated by the LINC complex (60,61). KO mouse studies have shown that nesprin-1 and SUN1/SUN2 play critical roles in anchoring nuclei in skeletal muscle (62,63). Similarly, mutations identified in *SUN1* and *SUN2* genes associated with EDMD like phenotypes cause defective nuclear positioning when expressed in mouse fibroblasts (15,16). The MT-based kinesin motor, kinesin-1, consisting of two KHCs and two KLCs, is localised at the NE. Binding of the dominant light chain isoform in muscle, KLC-2, to nesprin-2 has been shown to be responsible for nuclear rotations and movement along MTs (27). There is evidence that nuclear position can influence gene expression. For example, nuclei at the neuromuscular junction (NMJ) have a unique transcriptional profile relative to the non-synaptic nuclei (59). Nesprin-1 (*SYNE1*) levels are much higher in synaptic nuclei than extrasynaptic nuclei, and also higher in myotubes than myoblasts in culture and central nuclei during regeneration (52,64). In addition, maintaining proper nuclear positioning is thought to ensure sufficient transcriptional capacity and minimise transport distances between the nuclei and the cytoplasm in highly organised long muscle cells (60,65). Our data confirmed that nesprin-1 binds to KLC-1/2, and nesprin-1 mutants, particularly R8272Q, disrupted this interaction. Furthermore, depletion of KLC-1 resulted in nuclear clustering, whereas depletion of KLC-2, a dominant muscle isoform, caused reduction of MHC levels and the fusion index in myotubes, that was consistent with those observed in the R8272Q mutant. This disruption of nesprin-1/KLC interaction in C2C12 myoblasts may result in defects in nuclear movement along MTs, leading to abnormal nuclear positioning. Further investigations are required to elucidate the precise roles of nesprin-1 and KLC-1 and -2 in myonuclear positioning in muscle cell differentiation.

### Expression of nesprin-1α_2_ mutants induce less severe heart defects during zebrafish heart development compared to 1α_2_ WT

To investigate whether the nesprin mutants affect cardiac structure or function *in vivo*, we generated a zebrafish model by expressing human nesprin-1α_2_ WT and mutants (R8272Q, S8381C and N8406K) in zebrafish embryos via injection of the corresponding human SYNE1α_2_ (nesprin-1α_2_) mRNAs. Our data showed that human nesprin-1α_2_ WT causes heart developmental and conduction defects in zebrafish embryos, and all three 1α_2_ mutants caused less severe heart developmental defects. Although these results are unexpected, there are some potential explanations. Human nesprin-1α_2_ is not expressed in the heart and muscle until the initial stages of muscle cell differentiation (7,52,53). Therefore, injection of human nesprin-1α_2_ mRNA into zebrafish larvae at the one cell stage, when the expression level of endogenous nesprin-1 was minimal, caused ectopic expression effects on heart development. The effect of the mutants was less severe potentially because their binding interactions are compromised leading to less disruption. Future studies utilising the zebrafish model with either cardiac (tissue specific)/heat shock protein (timing dependent) promotors or CRISPR/Cas9-mediated knock-in is required to clarify underlying disease mechanism.

**Figure 10:**
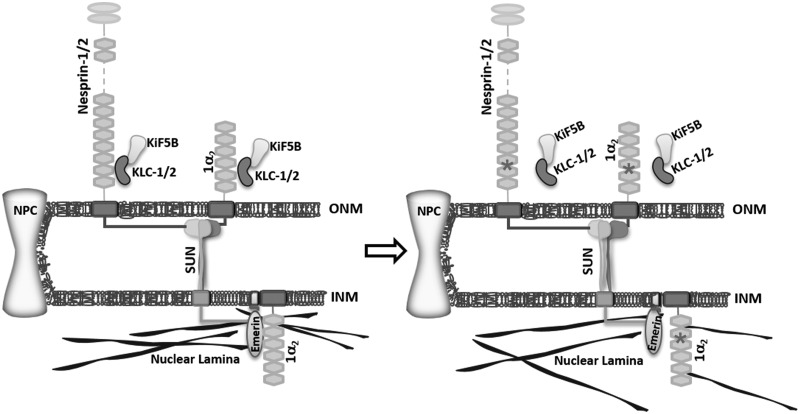
Working model for the role nesprin-1. The schematic figure shows nesprin-1 plays multiple-functions at both INM and ONM, and how mutants can disrupt the NE-LINC complex, contributing to the pathogenesis of muscle disease. *Indicates where the mutants are.

In summary, our data support the hypothesis that nesprin-1 plays multi-functional roles at both INM and ONM during muscle cell development and disease (Fig. 10). Although the role of nesprin-1 dysfunction in DCM requires further experimentation, we show that novel nesprin-1 mutants affect diverse functions, including gene expression and myoblast fusion and differentiation. Further investigation is now required to elucidate the complex mechanisms behind this dysregulation especially in the cardiac system.

## Materials and Methods

### Research subjects

The study cohort consisted of 218 unrelated individuals with DCM and 210 healthy controls, collected from the West China Hospital, Sichuan University, China. All blood materials of the patients and controls included in this study were taken with informed consent for DNA analysis and approval of the local ethics board. The clinical characteristic of the patients and controls were summarized in Supplementary Material, Table S1 and Supplementary material. Most of DCM patients were men and had significantly larger left ventricular (LV) chamber and lower left ventricular ejection fraction (LVEF) when compared with the ethnic and age-control samples.

### Mutation analysis

Primer pairs were designed across the intron/exon boundaries and untranslated regions of nesprin-1α_1_ and 1α_2_, nesprin-2α_1_, 2α_2_, 2β, 2ε_1_ and 2ε_2_, i.e. the smaller INM localised isoforms of nesprin-1 and nesprin-2 shown to bind emerin and lamin and be either highly or specifically expressed in muscle tissue. 180 oligonucleotide primers from intronic sequences for 88 exons were designed using the program Primer3Input (primer3_www.cgi v 0.2) and used previously (13). Mutation screening was performed using the PCR-based mutation detection technique DHPLC (WAVE 4500B system, Transgenomic). Appropriate DHPLC conditions for running temperatures and buffer gradients were established for each individual exon.

### Plasmid constructs/retroviral constructs/site-directed mutagenesis

Human cDNA of nesprin-1α_2_ WT constructs (amino acids 1–977, equivalent to nesprin-1 giant 7875–8796) were amplified using a high-fidelity GC-rich PCR kit (Roche) and inserted respectively in-frame into the EcoRI site of the pEGFP-C1 vector (Clontech) for overexpression of GFP-nesprin, and the NotI and EcoRI sites of pcDNA3.1(-) vector (Invitrogen) for *in vitro* synthesis of Flag-tagged nesprin mRNA and microinjection. GST nesprin-1α_2_ SR1-6 construct (amino acids 1–837, lacking KASH domain, equivalent to nesprin-1 giant 7875–8662, SR 69–74) and V5-tagged nesprin-1α_2_ construct (amino acids 1–977, equivalent to nesprin-1 giant 7875–8796) were amplified from the established pEGFP-C1-nesprin-1α_2_ WT construct and inserted into EcoRI and SalI sites of the pGEX-4T-1 vector (Pharmacia) and EcoRI and NotI sites of the MIGplus retroviral vector respectively. The MIGplus vector was a kind gift from Prof. Peter Zammit (66), which was modified from the retroviral (RV) backbone pMSCV-puro (Clontech, Mountain View, CA), in which the puromycin selection gene was replaced with eGFP to create pMSCV–IRES–eGFP, served as the RV control vector and *eGFP* as a reporter for retroviral infection. Retroviruses were then packaged into 293T cells using standard methods as described previously (66). All the constructs of nesprin-1 mutant constructs (R8272Q, S83831C, N8406K) were generated using QuickChange™ XL site-directed mutagenesis kit (Stratagene). Primers for the constructs above are listed in Supplementary Material, Tables S3–S5. Myc-SUN2 has been reported previously (67). CB6-HA-KCL2 WT and mutant N287L were kind gifts from Dr Mark Dodding (King’s College London, UK). GST-lamin A (amino acids 356–665) and GST-emerin (amino acids 1–176) were kind gifts from Dr Juliet Ellis (King’s College London, UK).

### Cell nuclei circularity quantification

‘Analyse particles’ function in ImageJ was used to measure circularity of cell nuclei, with circularity values given between 0 and 1 (values closer to 1 being more circular in shape) (68).

### Antibodies and immunofluorescence

Primary antibodies were sourced as follows: lamin A/C (N-18, sc-6215, Santa Cruz), SUN2 [kind gifts from Dr Didier Hodzic, University of Washington, US and (67)], emerin (NCL-EMERIN, Novacastra), GFP (ab290, ab13970, Abcam), GAPDH (sc-25778, Santa Cruz), β-actin (A5316, Sigma), α-tubulin (ab52866, Abcam), phospho-p44/42 MAPK (pERK1/2) (4370, Cell Signalling Technology), p44/42 MAPK (tERK1/2) (9102, Cell Signalling Technology), V5 (R96025, Invitrogen), nesprin-1 (MANNES1A and MANNES1E (52), generated against the C-terminus of the nesprin-1 giant), myogenin (sc-576, Santa Cruz), myosin (clone A4.1025, against all isoforms expressed by *MYH1*, Alexis Corporation), HA (ab1424, Abcam), KLC-1/2 (63–90, a kind gift from Prof Scott Brady, University of Illinois at Chicago, USA). Alexa fluorophore (488/546/647)-conjugated secondary antibodies were from Invitrogen. HRP-conjugated secondary antibodies were from Amersham. IF staining was performed as described previously (13). In particular, to further define the subcellular localisation for GFP-tagged nesprin-1α_2_, the transfected cells were fixed by 4% paraformaldehyde/PBS, then permeabilized using either 0.001% digitonin/PBS or 0.5% NP40/PBS.

### Cell culture, transfection and RNAi

HDFs, U2OS, C2C12 myoblasts were cultured at 37 °C/5% CO_2_ in Dulbecco’s modiﬁed Eagle’s medium (DMEM) supplemented with 10% fetal calf serum (FCS) and 1% Pen-Strep-Glutamine (PSG). For myoblast differentiation, C2C12 were cultured in low-mitogen medium (DMEM supplemented with 1% PSG and 2% horse serum) as described previously (7). For transient transfection, cells were plated onto glass coverslips or T25 flask at approximately 1.2 × 10^5^ cells/ml and transfected using FuGENE HD™ (Promega) according to the manufacturer’s instructions and fixed for IF staining or harvested for Western blotting 24 h after transfection as described previously (7,13). siRNA transfections were also performed by using HiPerFect (Qiagen) according to the manufacturer’s instructions. KLC-1/2 siRNA oligomers targeting to the mouse KLC-1/2 were previously described (27) and obtained from Dharmacon, named as KLC-1A and KLC-2A. Additional four KLC-1/2 siRNA oligos obtained from Qiagen (Cat. No: SI01085154, SI01085168, SI01083327 and SI01083341) were renamed as KLC-1B and -1C, KLC-2B and -2C respectively. Allstars negative control siRNA was also supplied from Qiagen.

### Neonatal rat cardiomyocytes (NRCs) isolation, culture and transfection

Primary cultures of NRCs were isolated from 1- or 2-day-old neonatal Sprague–Dawley rats. Hearts were collected and atria were removed. Hearts were then washed, excised, minced and enzymatically digested at 37 °C with ADS buffer [116 mmol/L NaCl, 20 mmol/L HEPES, 0.8 mmol/L NaH_2_PO_4_, 5.6 mmol/L glucose, 5.4 mmol/L KCl, 0.8 mmol/L MgSO_4_] containing collagenase (57.5 U/mL) and pancreatin (1.5 mg/mL). The suspension was pre-plated to remove contaminating cells, before being cultured on gelatin (Sigma) pre-coated 35 mm petri dishes with a density of 2 × 10^5^ cells/ml. Cells were allowed to adhere for 24 h, and then transfected using Escort III (Sigma) following the manufacturer’s instruction as described previously (69).

### Retroviral infection and myoblast differentiation

Retroviral constructs, together with an ectopic packaging plasmid, were transiently co-transfected into 293T cells to produce non-replicating retrovirus and the supernatant harvested 48, 60 and 72 h later. Retroviral infection was performed as described previously (66). Briefly, C2C12 cells were plated in T25 flasks. After 24 h, the medium was replaced with a 1:5 dilutions of 293T retroviral supernatant supplemented with 4 µg/ml polybrene and incubated at 37 °C for 4–6 h, and then changed into fresh medium. FACS was performed to purify the infected GFP positive cells in 48 h and these were used to in subsequent myoblast differentiation experiments. To induce differentiation, the medium was replaced with the differentiation medium (DM) containing 2% horse serum and 1% PSG, and cell lysates were collected for further investigation at day 0, 2, 4 and 6.

### Co-immunoprecipitation assays

U2OS cells were transfected using GFP-nesprin-1α_2_ WT/mutants with HA-KLC-1/2 WT/mutant as well as Myc-SUN2 respectively, harvested about 24 h later and kept in IP buffer [10 mM Tris (PH 7.4), 50 mM NaCl, 5 mM EDTA, 1% Triton X-100] with protease inhibitor cocktail (Sigma) on ice for 30 min, followed by 10 s sonication and centrifugation. About 500 µg of protein was pre-cleaned with Protein A/G sepharose beads (Sigma) for 1 h at 4 °C. Beads were removed by centrifugation and 2.5 µg primary antibody was added to lysates and left rotating at 4 °C for 16 h. Beads were then added to reactions and incubated for 2 h rotating at 4 °C. Samples were centrifuged and supernatant discarded. Pellets were washed and re-suspended in sample buffer, heated at 95 °C for 10 min and analysed by WB as described previously (13).

### GST pull-down assays

GST fusion proteins and GST alone were induced from 100 ml of bacterial culture for 4 h by addition of 0.2 mM isopropyl-1-thio-β-d-galactopyranoside. Purification of the proteins was performed according to the Amersham Biosciences protocol using glutathione-Sepharose 4B beads (Amersham Biosciences). Pull-down assays were performed as described previously (70). Briefly, 200 μg extracts from U2OS or C2C12 myoblasts/myotubes were incubated with 50 μl beads with constant rotation for 16 h at 4 °C. Bound proteins were washed and eluted into sample buffer, followed by WB. In particular, to investigate the interaction between nesprin-1 and its NE binding partners, protein lysates from Myc-SUN2 transfected (for SUN binding) and untransfected U2OS cells (for lamin A/C and emerin binding), C2C12 myoblast and myotubes (for KLC-1/2 interaction) were harvested and subjected to pull down using either GST-WT or mutant nesprin-1α_2_ SR1-6 beads. To further confirm the bindings, U2OS cells were transfected with either GFP-nesprin-1α_2_WT or mutants, protein lysates were harvested and subjected to pull-down using either GST-lamin A (amino acids 356–665) or GST-emerin (amino acids 1–176).

### Quantitative RT-PCR (QPCR)

C2C12 Cells were cultured in T25 flasks in proliferation or differentiation medium. Total RNA was extracted using RNA-STAT 60 (Amsbio) according to manufacturer’s protocol. Reverse transcription synthesis of cDNA and qPCR was performed as described previously (13). Relative expression of each myogenic transcriptional factor between proliferating and differentiated cells was measured in three replicates in at least three independent experiments. Primers for myogenin, MHC, MyoD, V5 and GFP have been previously described and listed in Supplementary Material, Table S6.

### Zebrafish embryos

WT embryos from AB strain were used. Embryos were obtained by natural matings and cultured in embryo medium (71). Staging of the embryos was carried out as described by Kimmel *et al*. (72). Ethical approval was obtained from the Animal Care and Use Committee of Sichuan University.

### *In vitro* synthesis of mRNA and microinjection

Capped GFP and nesprin mRNAs were synthesized using mMESSAGE mMACHINE^®^ Kit (Ambion); Synthetic capped mRNAs were injected into single-cell embryos. Injection dose (60 mg) was an optimised amount received by a single embryo.

### Zebrafish whole-mount *in situ* hybridization

Whole-mount *in situ* hybridization was carried out as previously described (73,74). After linearization by appropriate restriction enzymes, antisense RNAs for *in situ* hybridization were synthesized using DIG RNA Labelling Kit (SP6/T7) (Roche) and purified by MEGAclear (Ambion). Signal area of whole-mount *in situ* hybridization was measured by software ImageJ.

### Statistical analysis

Cell counts for statistical analysis were performed on *n* = 100–200 cells/*n* = 10 confocal microscope fields (63× magnification) for each control and experimental group, and results were verified in at least three independent experiments. The data were analysed using GraphPad Prism software by the Student’s *t*-tests, One way or Two-way analysis of variance (ANOVA) with Dunnett’s multiple comparison test for two independent groups or multiple comparisons, respectively. The values are expressed as mean ± standard error of mean (SEM). The *P*-values < 0.05 were considered statistically significant.

## Supplementary Material

Supplementary Material is available at *HMG* online.

## Supplementary Material

Supplementary Data
