# Supplementary material for: Novel nesprin-1 mutations associated with dilated cardiomyopathy cause nuclear envelope disruption and defects in myogenesis
Source: Hum Mol Genet. 2017 Apr 7;26(12):2258–76. doi: 10.1093/hmg/ddx116 (PMC5458344; doi:10.1093/hmg/ddx116)
Supplement: Supplementary Data [file ddx116_Supp.zip › Zhou C et al - supplementary file1.docx]

**Supplementary Material**

***Supplementary Table 1.*** ***Clinical features of research subjects***

|  | DCM patients  (n=218) | Healthy controls  (n=210) | p-value |
| --- | --- | --- | --- |
| Gender, % male | 67.0 (158/218) | 51.0 (117/210) | 0.0007 |
| Age at diagnosis/inclusion, year | 48.3±15.2 | 49.2±9.2 | 0.4565 |
| LVEDD, mm/m^2^ | 66.3 ±9.1 | 45.1 ± 5.9 | <0.0001 |
| LVEF, % | 33.2 ±13.3 | 59.3 ± 8.9 | <0.0001 |

Clinical features refer to the time of presentation. LVEDD, left ventricular end diastolic diameter;

LVEF, left ventricular ejection fraction.

***Supplementary Table 2.*** ***Rare (<1%) nesprin-1 variants identified in DCM patients***

| Exon | DNA variation | Amino acid  exchange | Allele frequency  in patients (%, n=436 alleles) | Allele frequency in a reference  population (%, n=420 alleles) |
| --- | --- | --- | --- | --- |
| 136 | c. 25417 G>A | **p. R8272Q** | 0.46 | 0 |
| 139 | c. 25743 A>T | **p. S8381C** | 0.46 | 0 |
| 139 | c. 25820 C>A | **p. N8406K** | 0.69 | 0 |
| 133 | c. 24299+12 G>A | intron | 0.69 | 0 |
| 135 | c.24308+3 A>G | intron | 0.46 | 0 |
| 136 | c.25463 C>T | p.H8287H | 0.92 | 0 |
| 139 | c.25730 G>C | p.L8376L | 0.69 | 0 |
| 139 | c.25748 C>T | p.S8382S | 0.46 | 0 |

GenBank reference sequence: nesprin-1 giant (*SYNE-1*): AF495910.1; 1α_2_: AY184203.1. Nucleotide and amino acid numbers are based on nesprin-1 giant. n, number of alleles analysed.

***Clinical description of the patients harbouring the mutations identified in this study***

### Patient No.1 was a male carrying R8272Q mutation. He had no family history of cardiovascular diseases and suffered shortness of breath after exertion since his forties. He was diagnosed with DCM and started receiving drug therapy including diuretics and digoxin since the age of 59. His symptom exaggerated at the age of 68, echocardiography showed the LV diameter was 90mm with very thin interventricular septum (IVS) and left ventricular posterior wall (LVPW). LVEF was 26%. N-terminal pro [B-type Natriuretic Peptide](http://my.clevelandclinic.org/services/heart/diagnostics-testing/laboratory-tests/b-type-natriuretic-peptide-bnp-bloodtest) (pro-BNP) was 2912 pg/ml, significantly greater than normal reference value (0-227 pg/ml). ICD was subsequently implanted. Patient died of progressive left-sided heart failure one year later.

Patient No.2 was a male carrying R8272Q mutation. He has no family history of cardiovascular diseases. He suffered shortness of breath after exertion since the age of 52, one year later he was diagnosed with DCM.  Echocardiography showed the LV diameter was 72mm, LVEF was 37%.

### Patient No.3 was a male carrying S8381C mutation. He has no family history of cardiovascular diseases. He suffered shortness of breath after exertion at the age of 56, the LV diameter was 56 and LVEF was 34%. ECG showed sinus rhythm, pro-BNP level was 1847 pg/ml, greater than normal reference value. He was treated with beta-blocker, diuretics and digoxin and then free of symptom.  At the age of 62, echocardiography showed the LVEF was 24%. ECG showed atrial fibrillation (AF), pro-BNP increased to 4140 pg/ml.

Patient No. 4 was a male carrying N8406K mutation. He has no family history of cardiovascular diseases. His first symptom was repeated palpitation. At the age of 67, he began to feel fatigue after exertion. Ambulatory electrocardiogram revealed ventricular tachycardia. Echocardiography showed his LV diameter was 67mm and LVEF was 42%. He refused ICD implantation and thus was treated with beta-blocker and amiodarone.

### Patient No. 5 was a male carrying N8406K mutation. He has no family history of cardiovascular disease. He suffered shortness of breath after exertion at the age of 68, ECG showed AF. He was treated with beta-blocker, diuretics and digoxin. He died of cardiac arrest at the age of 68. The diagnosis of DCM was further confirmed by autopsy.

No clinical data was available for patient No. 6 (S8381C) and No.7 (N8406K).

*Supplementary Table 3. Primers used in site-directed mutagenesis for Nesprin-1α_2_*

| Missense mutations | Primers | |
| --- | --- | --- |
| R8272Q | Forward | 5’ CGGTCAGGAC**A**AGACACCCCA 3’ |
|  | Reverse | 5’ TGGGGTGTCT**T**GTCCTGACCG 3’ |
| S8381C | Forward | 5’ GCTGGGCGAATGC**T**GTAGCAGTATAGA 3’ |
|  | Reverse | 5’ TCTATACTGCTAC**A**GCATTCGCCCAGC 3’ |
| N8406K | Forward | 5’ CTGGCTTTGTTAA**A**CTGCATAGTACCG 3’ |
|  | Reverse | 5’ CGGTACTATGCAG**T**TTAACAAAGCCAG 3’ |

The mutated nucleotides are in bold.

##### ***Supplementary Table 4. Primers for generating V5-tagged nesprin-1α****­_2_* ***WT and -1 KASH***

| Name | Primers | |
| --- | --- | --- |
| nesprin-1α­_2_ WT | Forward | 5’ATAGCGGCCGCCACCATGGGAAAGCCTATTCCTAATCCTCTTCTAGGTCTAGATTCTACTGGCGGAGGCGTGGTGGCGGAGGAC 3' |
| nesprin-1 KASH | Forward | 5’ATAGCGGCCGCCACCATGGGAAAGCCTATTCCTAATCCTCTTCTAGGTCTAGATTCTACTGGCGGAGGCCGCGGCTTCCTGTTCA 3’ |
| nesprin-1α­_2_ WT/KASH | Reverse | 5’ TATAGAATTCTCAGAGTGGAGGAGGGCCATTC 3' |

##### ***Supplementary Table 5. Primers for generating Flag-tagged nesprin-1α****­_2_* ***WT***

| Name | Primers | |
| --- | --- | --- |
| nesprin-1α­_2_ WT | Forward | 5’ATAGCGGCCGCCACCATGGACTACAAAGACGATGACGACAAGGGCGGAGGCGTGGTGGCGGAGGACCTG 3' |
| nesprin-1α­_2_ WT | Reverse | 5’ TATAGAATTCTCAGAGTGGAGGAGGGCCATTC 3' |

***Supplementary Table 6.*** ***Primers used for qPCR***

| Name | Primers | |
| --- | --- | --- |
| Myogenin | Forward | 5’ TGTTTGTAAAGCTGCCGTCTGA 3’ |
|  | Reverse | 5’ CCTGCCTGTTCCCGGTATC 3’ |
| MHC | Forward | 5’ AGAGCTGACGTGCCTCAATG 3’ |
|  | Reverse | 5’ ATGCCTCTTCTTGCCCTTGT 3’ |
| MyoD | Forward | 5’ GCCGCCTGAGCAAAGTGAATG 3’ |
|  | Reverse | 5’ CAGCGGTCCAGGTGCGTAGAAG 3’ |
| V5 | Forward | 5’ ATATCCTCGTTCGACCCCGCC 3’ |
|  | Reverse | 5’ TCATTAAGCTTTCTCTGTATTTCTTCCGAG 3’ |
| GFP | Forward | 5’ GGTCGAGCTGGACGGCGACGTAAA 3’ |
|  | Reverse | 5’ CACCAGGGTGTCGCCCTCGAACTT 3’ |

**Legend to Supplementary video:**

The representative live images showed slow heart rate and dilated atrial chambers for the embryo injected with nesprin-1α_2_ WT mRNA, and also abnormal body development for the affected embryos.
