# Supplementary material for: Novel nesprin-1 mutations associated with dilated cardiomyopathy cause nuclear envelope disruption and defects in myogenesis
Source: Hum Mol Genet. 2017 Apr 7;26(12):2258–76. doi: 10.1093/hmg/ddx116 (PMC5458344; doi:10.1093/hmg/ddx116)
Supplement: Supplementary Data [file ddx116_Supp.zip › HMG-2017-D-00138 Zhou C et al -supplementary file2.pdf]

Supplementary Fig .1

A.

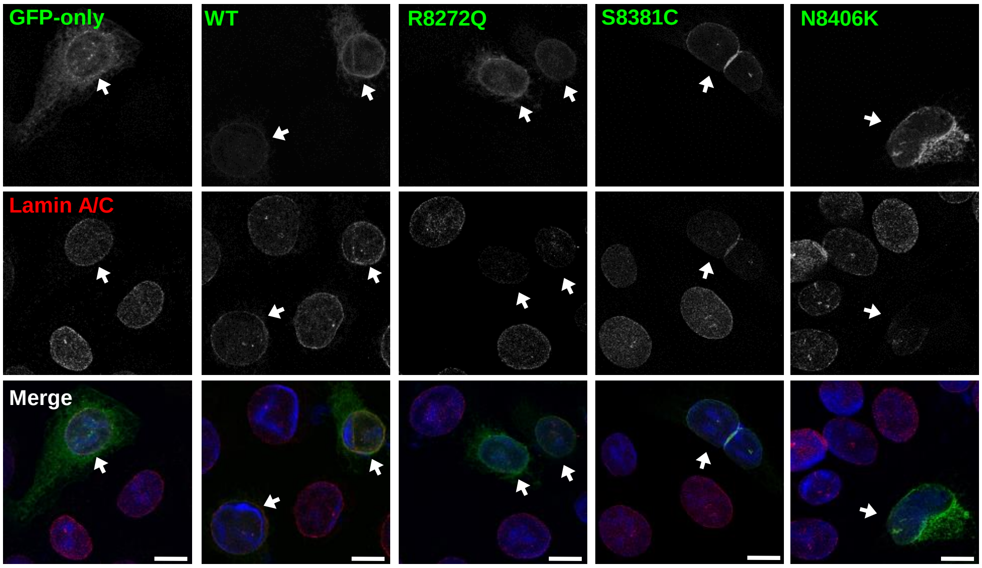

Scale bar=12.5 μm

B.

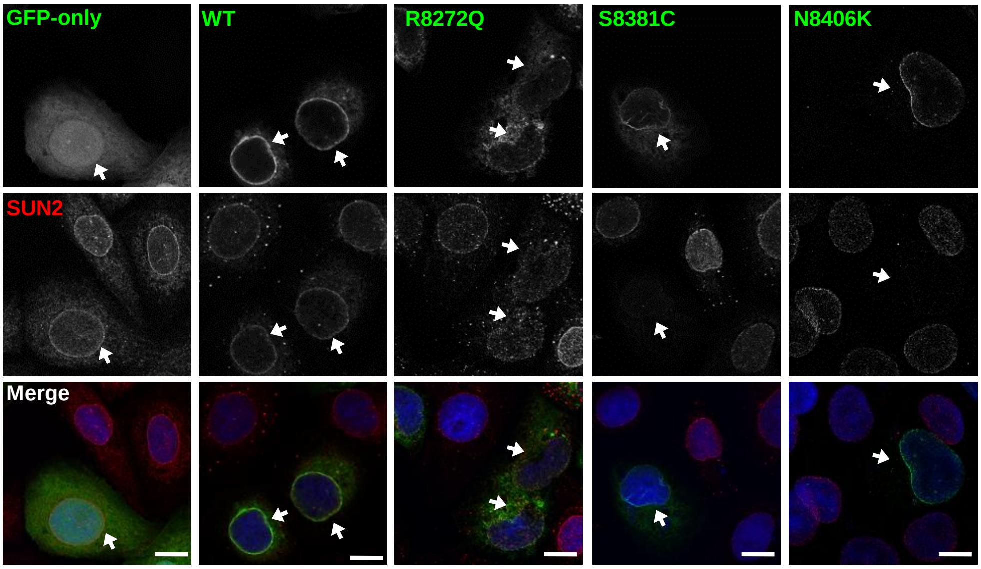

Scale bar=12.5 μm

IF showed exogenous expression of all three novel nesprin-1 mutants, especially S8381C, caused weaker staining of lamin A/C at the NE (A), while all three mutants caused weaker staining of SUN2 at the NE (B) when compared with a WT nesprin-$1\alpha_2$ construct in U2OS cells.

Supplementary Fig .1

C.

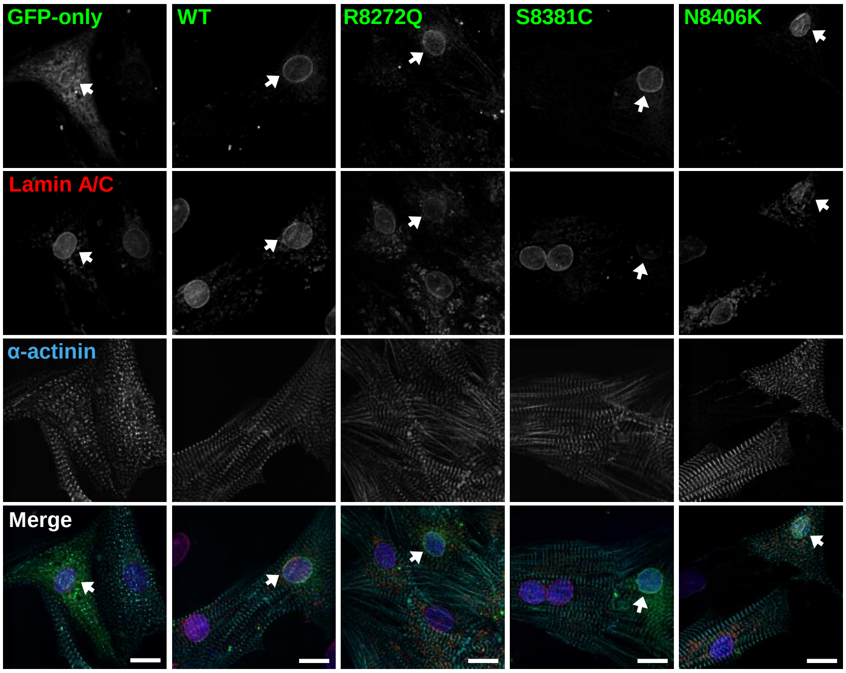

Scale bar=15 µm

D.

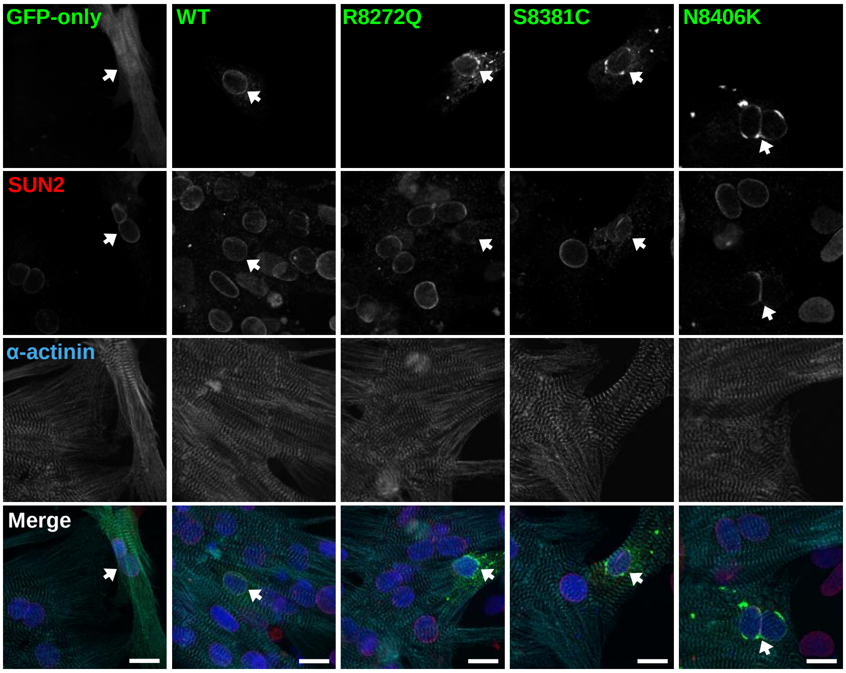

Scale bar=15 µm

IF showed both lamin A/C (C) and SUN2 (D) staining was weaker at the NE with all three mutants compared with WT nesprin-1α_2 in NRCs.

Supplementary Fig .2

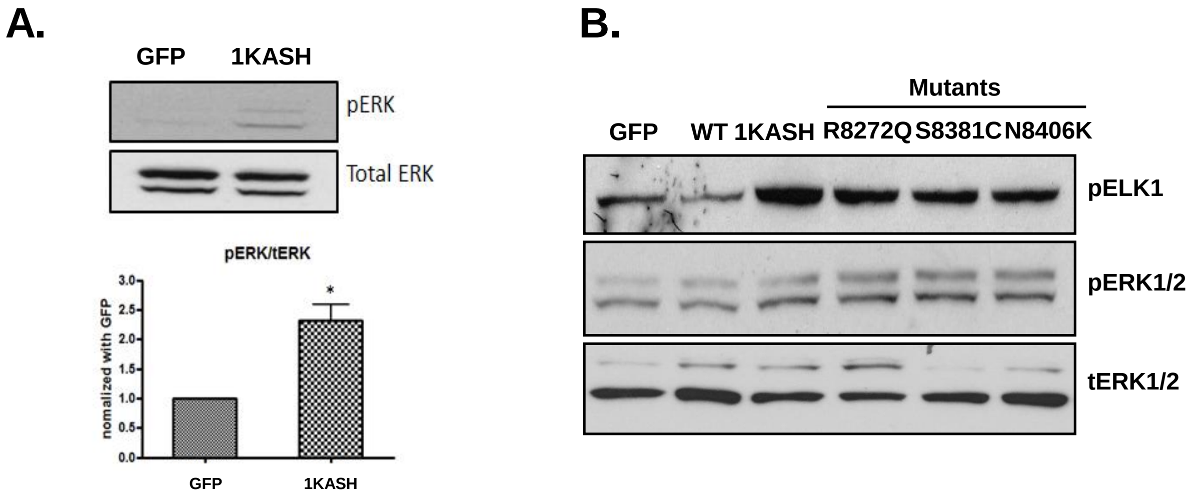

Overexpression of dominant negative nesprin-1 KASH led to aberrant activation of ERK pathway (A), and three mutants led to increase pERK1/2 and pELK1 activities (B).

Supplementary Fig .3

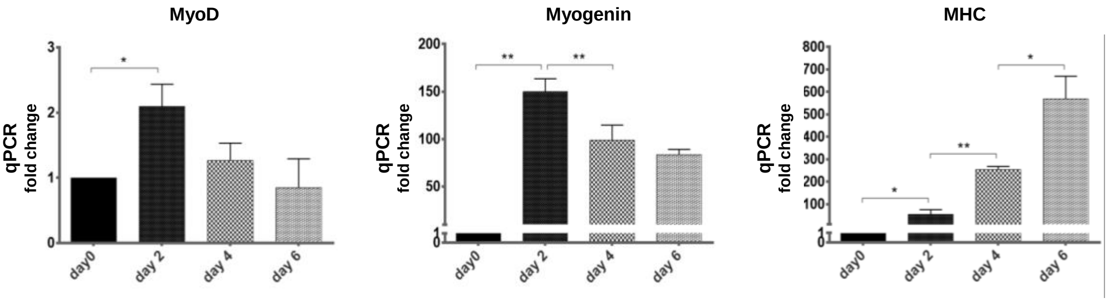

qPCR showed endogenous expression levels MyoD, myogenin and myosin increased during myotube formation. MyoD and myogenin levels peaked at day 2. MHC was detected from day 2 and increased until day 6.

Supplementary Fig .4

A. B.

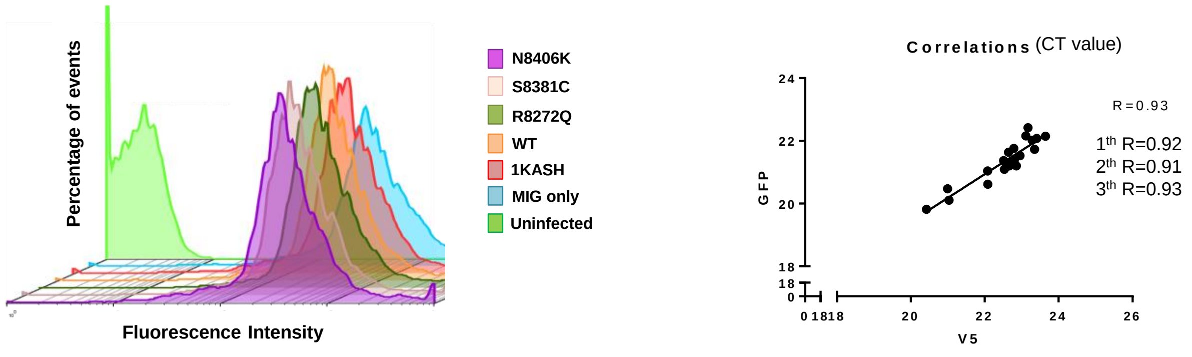

Retroviral V5-tagged nesprin-1α_2 WT/mutants and dominant negative-1KASH were generated in a MIG (IRES-GFP) retroviral vector and infected into C2C12 myoblasts respectively. FACS was performed to purify the infected GFP positive populations and these were used in subsequent muscle cell differentiation experiments (A). qPCR showed positive correlation of the expression levels (CT value) between GFP and V5-nesprin1α_2 (B).

Supplementary Fig .5

A.

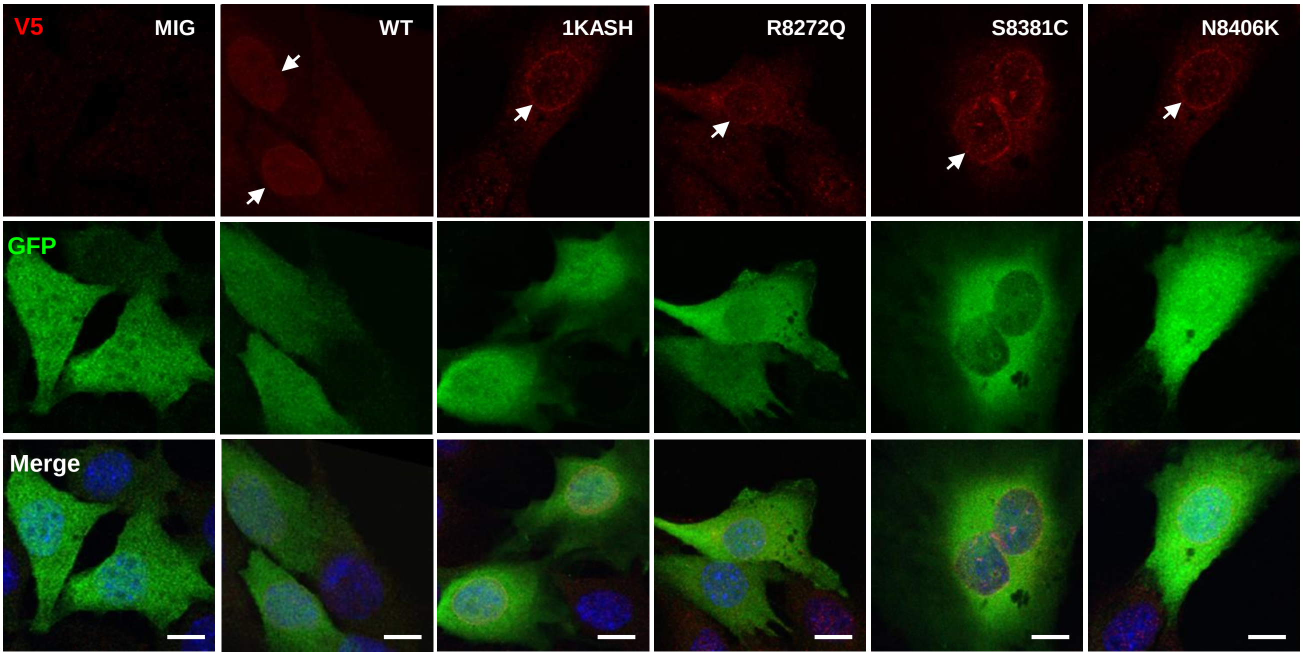

Scale bar=25 μm

B.

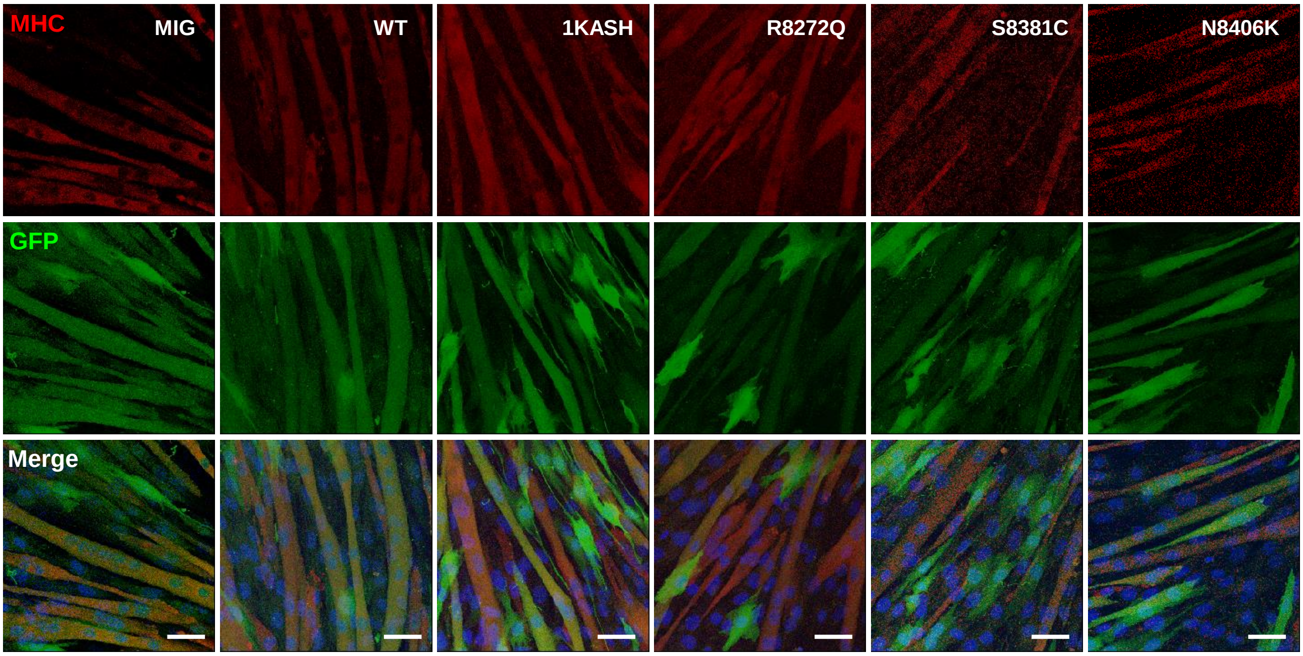

Scale bar=50 μm

IF showed that exogenous expressed V5-tagged $1\alpha_2$ WT or mutants and dominant negative-1KASH were localised at the NE (A). Upon differentiation, fewer multinucleated myotubes were observed in the cells transduced with three mutants and dominant negative-1KASH domain compared with the C2C12 cells transduced with GFP alone (MIG only) or nesprin-$1\alpha_2$ WT .

Supplementary Fig .6

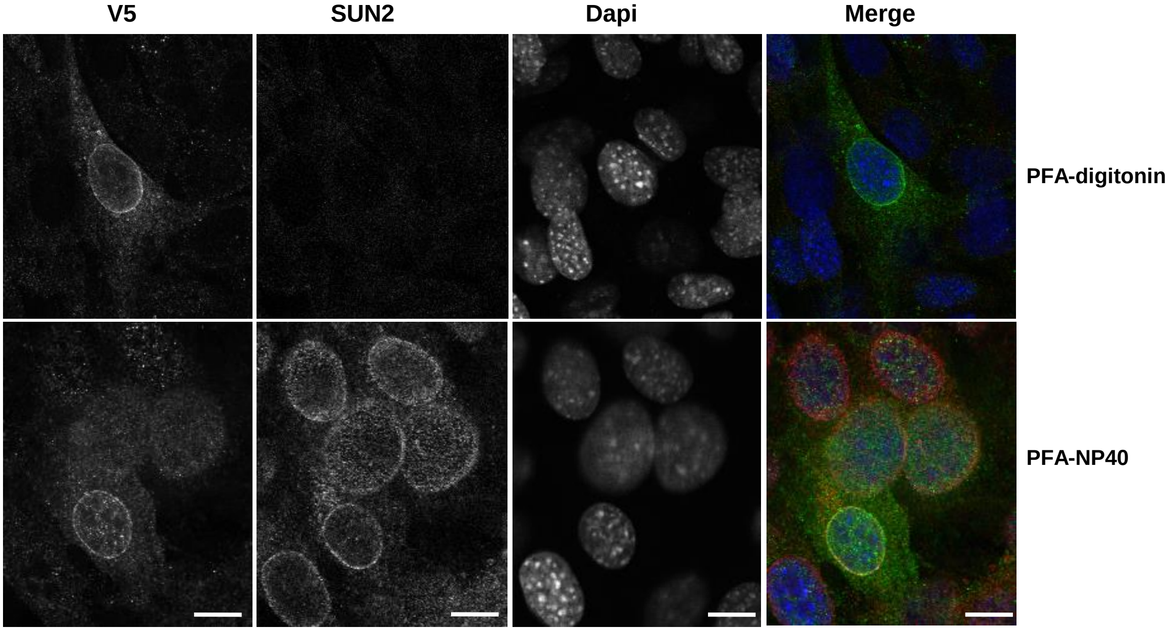

Scale bar=12.5 µm

IF staining showed that V5-nesprin-1α_2 was present at both ONM (digitonin treatment) and INM (NP40 treatment).

Supplementary Fig .7

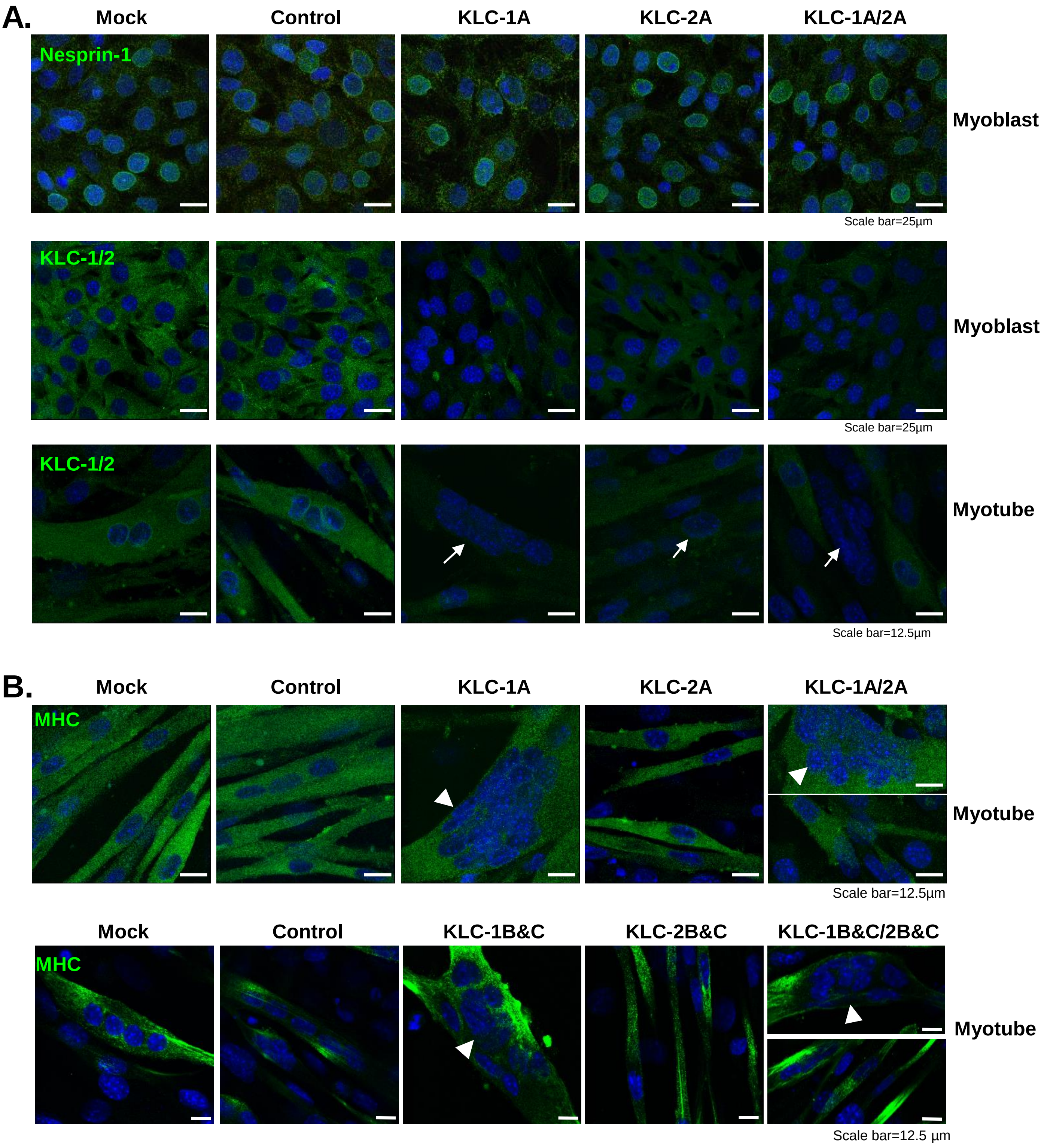

KLC-1/2 depletion caused defects in myoblast fusion and differentiation. IF showed KLC-1/2 staining at the NE (arrowed) was reduced in myotubes, but nesprin-1 staining (detected by MANNES1A) remained at the NE(A), and the nuclei aggregation and clustering (arrow-headed) were observed upon KLC-1 and -1/2 knockdown (B).

Supplementary Fig .7

C.

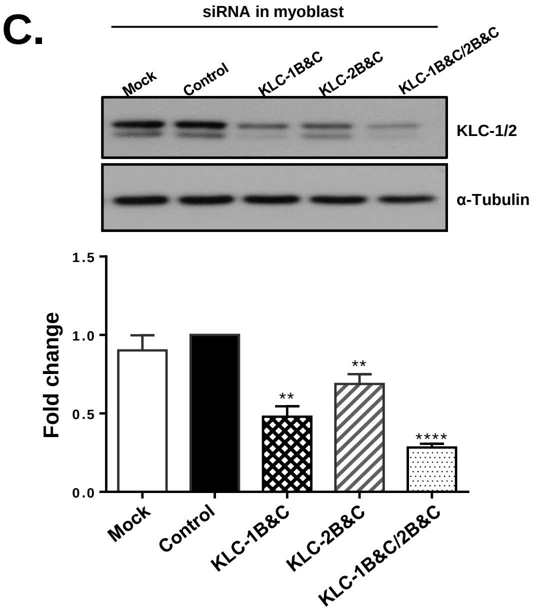

D.

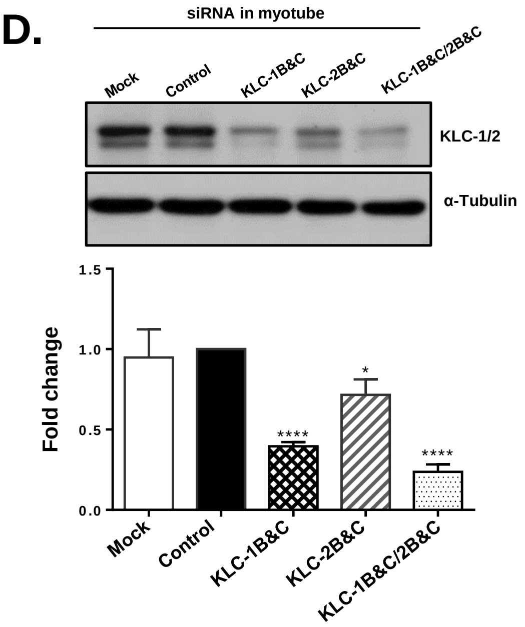

E.

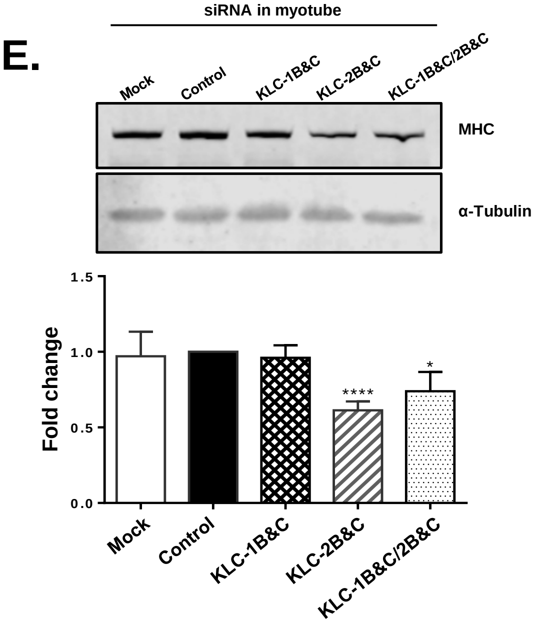

F.

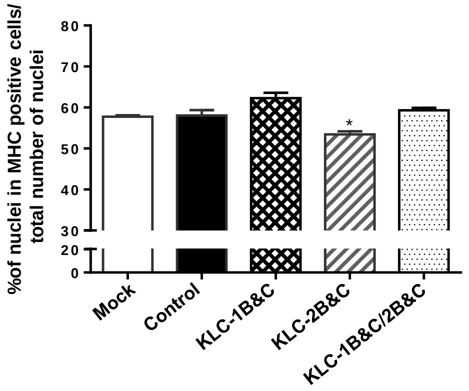

G.

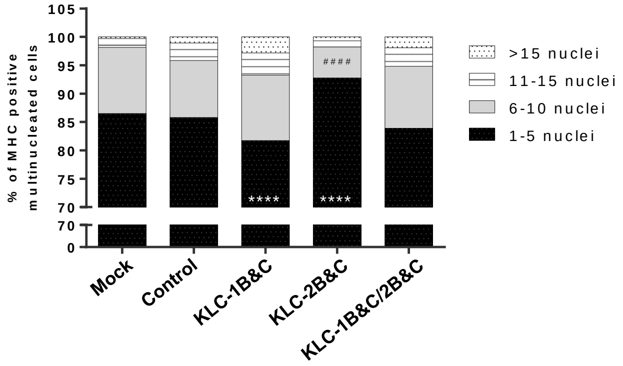

siRNA knockdown of KLC-1/2 caused defects in myoblast fusion and differentiation. WB showed the expression levels of both KLC-1 and -2 were reduced in myoblasts (C) and myotubes (D) upon KLC-1/2 depletion using either KLC-1B&C or/both KLC-2B&C oligos. The expression level of MHC (E) and the fusion index (F) were significantly reduced in myotubes especially upon KLC-2 depletion (using KLC-2B&C oligos), more than 800 nuclei for each clone were counted under microscopy (63x objective). Further analysis of MHC positive multinucleated cells revealed that KLC-2 depletion resulted in fewer nuclei per myotube (G). Means and SEM were obtained from three independent experiments for each clone. *p<0.05 using Student's *t*-tests or two-way ANOVA analysis.

Supplementary Fig .8

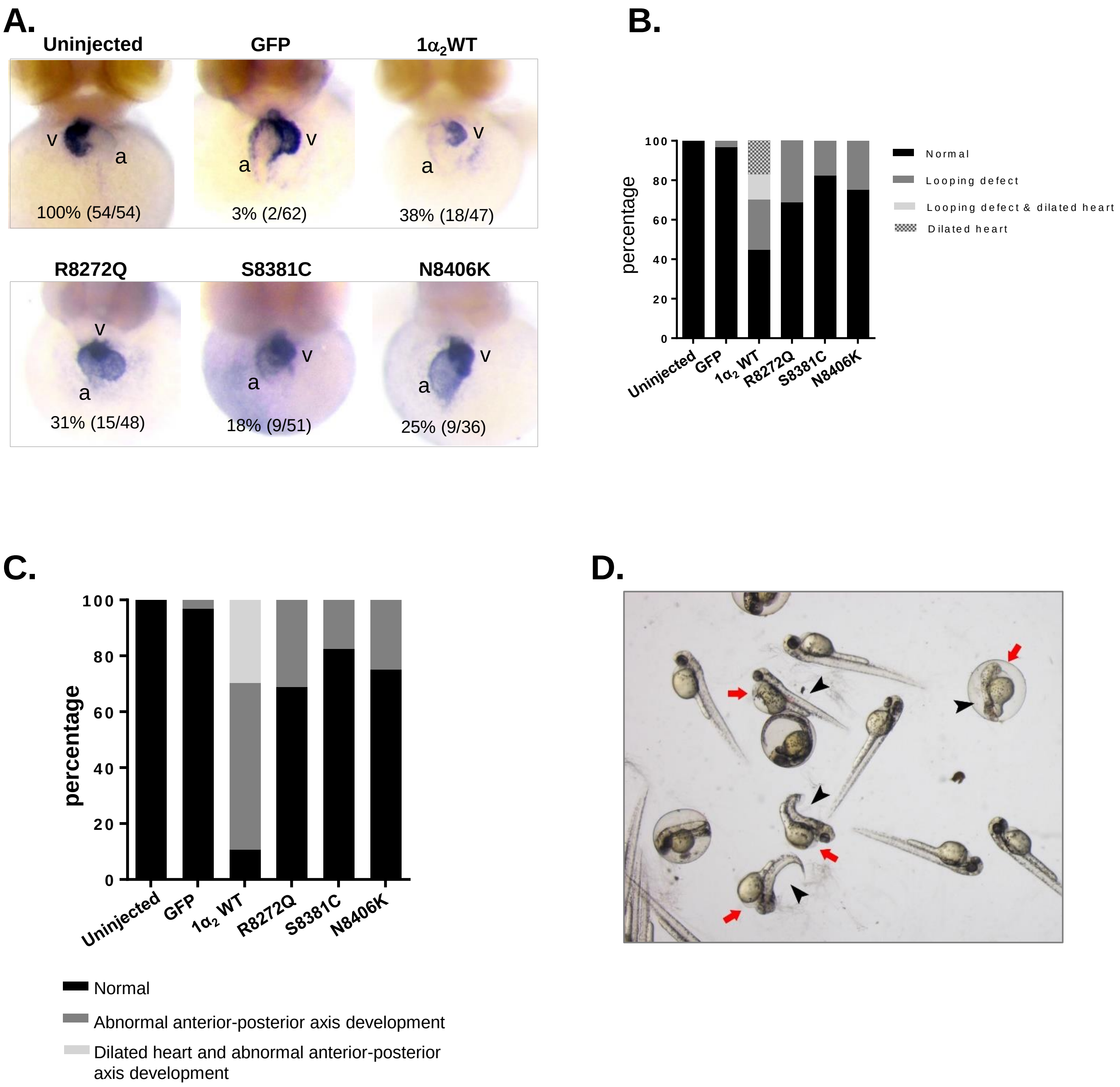

Human nesprin-1α_2 WT induced heart development defects while mutants induce a less severe heart phenotype in zebrafish embryos . WISH monitoring expression of the myl7 gene at 48 hpf (A), the numbers (left in brackets) indicate the percentage of embryos displaying the phenotype represented in the picture shown, the numbers (right in brackets) are the total numbers counted of observed embryos. Human nesprin-1α_2 WT caused a dilated heart phenotype (B-D), and both WT and mutants caused defects in heart looping when compared with uninjected shown (A,B) and abnormal anterior-posterior axis development (C, D) in the injected embryos when compared with uninjected and GFP expressing embryos. Approximately 36 to 62 embryos for each injection were counted; the representative picture (D) shows abnormal anterior-posterior axis development (black arrowhead) and dilated heart (red arrow) for the affected embryos.
